# Eating Disorders: An Evolutionary Psychoneuroimmunological Approach

**DOI:** 10.3389/fpsyg.2019.02200

**Published:** 2019-10-29

**Authors:** Markus J. Rantala, Severi Luoto, Tatjana Krama, Indrikis Krams

**Affiliations:** ^1^Department of Biology, University of Turku, Turku, Finland; ^2^English, Drama and Writing Studies, University of Auckland, Auckland, New Zealand; ^3^School of Psychology, University of Auckland, Auckland, New Zealand; ^4^Department of Biotechnology, Daugavpils University, Daugavpils, Latvia; ^5^Institute of Ecology and Earth Sciences, University of Tartu, Tartu, Estonia

**Keywords:** anorexia nervosa, binge eating, bulimia nervosa, evolutionary psychiatry, neuroinflammation, stress responsivity, mismatch hypothesis, adaptive metaproblem

## Abstract

Eating disorders are evolutionarily novel conditions. They lead to some of the highest mortality rates of all psychiatric disorders. Several evolutionary hypotheses have been proposed for eating disorders, but only the *intrasexual competition hypothesis* is extensively supported by evidence. We present the *mismatch hypothesis* as a necessary extension to the current theoretical framework of eating disorders. This hypothesis explains the evolutionarily novel *adaptive metaproblem* that has arisen when mating motives conflict with the large-scale and easy availability of hyper-rewarding but obesogenic foods. This situation is exacerbated particularly in those contemporary environments that are characterized by sedentary lifestyles, ever-present junk foods, caloric surplus and the ubiquity of social comparisons that take place via social media. Our psychoneuroimmunological model connects ultimate-level causation with proximate mechanisms by showing how the adaptive metaproblem between mating motives and food rewards leads to chronic stress and, further, to disordered eating. Chronic stress causes neuroinflammation, which increases susceptibility to OCD-like behaviors that typically co-occur with eating disorders. Chronic stress upregulates the serotonergic system and causes dysphoric mood in anorexia nervosa patients. Dieting, however, reduces serotonin levels and dysphoric mood, leading to a vicious serotonergic-homeostatic stress/starvation cycle whereby cortisol and neuroinflammation increase through stringent dieting. Our psychoneuroimmunological model indicates that between-individual and within-individual variation in eating disorders partially arises from (co)variation in gut microbiota and stress responsivity, which influence neuroinflammation and the serotonergic system. We review the advances that have been made in recent years in understanding how to best treat eating disorders, outlining directions for future clinical research. Current evidence indicates that eating disorder treatments should aim to reduce the chronic stress, neuroinflammation, stress responsivity and gut dysbiosis that fuel the disorders. Connecting ultimate causes with proximate mechanisms and treating biopsychosocial *causes* rather than manifest symptoms is expected to bring more effective and sophisticated long-term interventions for the millions of people who suffer from eating disorders.

## Introduction

Eating disorders are severe mental disorders with a biopsychosocial pathogenesis and a cost of about €1 trillion per year in the EU alone (Schmidt et al., 2016). They can become chronic and debilitating and are associated with significantly increased mortality rates (Schmidt et al., 2016). Anorexia nervosa, for example, has the highest mortality rate of all psychiatric disorders (5.10 deaths per 1,000 affected individuals: Arcelus et al., 2011). It is well known that the treatment of eating disorders is ineffective compared with the treatment of other mental disorders (Arcelus et al., 2011; Murray et al., 2019). This inefficiency is highlighted by the fact that current treatments are focused on reducing symptoms rather than treating the underlying cause(s) of eating disorders. The main reason for the ineffectiveness of existing treatments is, frankly, that the etiology of eating disorders is not properly understood (van Furth et al., 2016; Frank et al., 2019; Murray et al., 2019). Advances in the scientific knowledge of eating disorders are urgently needed.

Complete analyses of a trait or a behavior are ideally provided on two different but complementary levels: (1) what is the *proximate mechanism* underlying the trait: how does it work? – and (2) what is the *ultimate reason* it evolved: what fitness benefit, if any, does it provide for the organism? (Bateson and Laland, 2013; Rantala et al., 2018; Luoto et al., 2019a). We integrate these two levels of analysis and argue that without understanding both proximate mechanisms and ultimate causes, it is challenging to prevent eating disorders and to find effective treatments for them.

The fifth edition of the Diagnostic and Statistical Manual of Mental Disorders (DSM-5) specifies three eating disorders: anorexia nervosa (AN), bulimia nervosa (BN) and binge eating disorder (BED). In addition to these three disorders, DSM-5 recognizes the importance of subthreshold and atypical conditions by naming five specific Other Specified Feeding or Eating Disorder (OSFED) subtypes:

1. Atypical Anorexia Nervosa (i.e., anorexic features without low weight);
2. Bulimia Nervosa (of low frequency and/or limited duration);
3. Binge Eating Disorder (of low frequency and/or limited duration);
4. Purging Disorder;
5. Night Eating Syndrome.

DSM-5 also includes a category called Unspecified Feeding or Eating Disorder (UFED) that includes persons who do not fit into any of these five categories, or for whom there is insufficient information to make a specific OSFED diagnosis (American Psychiatric Association, 2013).

Several evolutionary hypotheses have been suggested for explaining eating disorders. We critically review these ultimate-level hypotheses (section “Existing Evolutionary Psychological Hypotheses for Eating Disorder”) and synthesize them with a novel proximate explanation of the physiological mechanisms underlying eating disorders (section “A Psychoneuroimmunological Model of Eating Disorders”). Our psychoneuroimmunological model suggests that eating disorders are not separate diseases – instead, they form a continuum. Based on variation in patients’ biobehavioral states, the continuum model explains why “unspecified eating disorders” is a common diagnosis (11–50.8% of the cases: Machado et al., 2013; Caudle et al., 2015; Mancuso et al., 2015) and why patient diagnoses may shift between eating disorders over time (section “Sources of Individual Differences in Responses to Intrasexual Competition”). The model provides an explanation for the finding that eating disorders are often comorbid with other mental disorders (section “Comorbidity of Eating Disorders”): according to our model, this shared transmission is mediated by vulnerability to neuroinflammation and stress responsivity (section “Risk Factors for Eating Disorders”). Our psychoneuroimmunological model leads us to suggest treatments (sections “Eating Disorder Treatments Based on Psychoneuroimmunology” and “Ultimate-Level Prevention of Eating Disorders”) informed by a synthetic understanding of both proximate mechanisms and ultimate causes. These treatments have the potential to offer significant advances on current eating disorder treatments, which are reviewed in section “Current Eating Disorder Treatment”.

## Existing Evolutionary Psychological Hypotheses for Eating Disorders

There are six existing evolutionary hypotheses for the ultimate causation of eating disorders. The leading evolutionary hypothesis for BN and BED is (1) the *thrifty genotype hypothesis*. It suggests that binge eating is a psychological adaptation (see e.g., Lewis et al., 2017 for a discussion of psychological adaptations) which arose because extra energy stores were protective in the evolutionary history of our species: they helped to avoid malnutrition, helped survival during famines and regulated reproduction (Chakravarthy and Booth, 2004; Wells, 2006). In an extension of this hypothesis, the *dual intervention point* model posits that the body has upper and lower set points for the level of body adiposity; if these are exceeded, physiological feedback mechanisms are triggered (Speakman et al., 2011; Speakman, 2018). The minimum set point for adiposity is needed to avoid starvation, while the maximum set point is determined by risk of predation. As the risk of predation has declined, genes coding for higher maximum set point have become more common, and fewer people reduce caloric intake to prevent weight gain (Speakman et al., 2011).

(2) The *intrasexual competition hypothesis* (Abed, 1998) suggests that the ultimate cause of eating disorders is intense intrasexual competition for mates. This hypothesis recognizes that a woman’s body shape is an indicator of her reproductive history, reproductive potential and mate value, partly signaled by waist-to-hip ratio and body mass index (BMI) (Andrews et al., 2017; Del Zotto and Pegna, 2017). As women age and/or reproduce, they tend to gain body mass and lose the hourglass body shape (Butovskaya et al., 2017) which is a sexually desirable trait for men (e.g., Bovet, 2019). Women’s reproductive window is finite, which is why men have evolved a preference for cues of fertility and youth (Sohn, 2016; Lassek and Gaulin, 2019). This can lead to women competing with each other for the attention of men by appearing to be youthfully slim: women’s reproductive value, after all, is associated with youthfulness, and youthfulness is associated with slimness (Abed, 1998; Lassek and Gaulin, 2019).

The intrasexual competition hypothesis is in line with studies showing that men’s preference for women’s bodies can vary from one environment and society to another (cf. Furnham and Baguma, 1994; Tovee et al., 2006). Plumpness may be an indicator of higher fertility in countries where malnutrition is common; in well-nourished populations, in contrast, relative plumpness is associated with aging and reduced fertility (e.g., Tovee et al., 2006). The intrasexual competition hypothesis suggests that the increased prevalence of eating disorders in westernized societies is a result of intensified intrasexual competition among women and/or the relative abundance of food (Abed et al., 2012; Baumeister et al., 2017; Nettersheim et al., 2018).

There are a number of factors that intensify intrasexual competition (Abed et al., 2012): (a) decline in fertility leads to an increased preservation of a nubile appearance in older women; (b) in modern Western societies, women have a higher ability to regulate their reproductive behavior (with minimal interference from kin); (c) there is an unusually high number of youthful and youthful-looking women, i.e., potential competitors, in modern cities as compared with ancestral conditions of humans; (d) media provides images of attractive competitors; (e) food is abundant and populations are well nourished, so weight gain and the deterioration of the nubile shape are typical concomitant features of advancing age; (f) the increasing instability of marriages and prevalence of divorces has led to both men and women to return repeatedly to the mating market; (g) due to the advent of modern medicine and increased life expectancy (i.e., increased number of post-menopausal women), youth has become one of the primary determinants of female mate value (reviewed in Abed et al., 2012; see also Baumeister et al., 2017; Saunders and Eaton, 2018; Lassek and Gaulin, 2019; Luoto, 2019a).

A study conducted in 26 countries with over 7,000 participants found that a thin body shape was preferred in areas with a high socioeconomic standing and that media exposure has a significant association with body weight ideal (Swami et al., 2010). Furthermore, Swami et al. (2010) found that women consistently thought that thinner female figures are more attractive than what men thought. Baumeister et al. (2017) reported that the more women perceived the local mating market to have a shortage of men, the more they wanted to be thin and the more they had signs of body dissatisfaction. These findings support the idea that intrasexual competition among women drives women’s pursuit of thinness.

More support for the hypothesized role of intrasexual competition behind eating disorders comes from “reverse anorexia” that affects male bodybuilders (Pope et al., 1993) and can drive body dissatisfaction more generally in boys and men (Karazsia et al., 2017). Affected individuals express the belief that they are too small despite being muscular, therefore having a distorted body image. Muscularity and large body size offer clear advantages in male-male competition in humans as in other mammals; muscularity may have also been a sexually attractive trait for women in our evolutionary history, with sexual selection acting on the trait even in present-day men (Frederick and Haselton, 2007; Sell et al., 2017).

Due to the intensified competition and the other evolutionarily novel factors mentioned above, most women in the Western world are dissatisfied with their body size and shape, with half of teenaged girls trying to control their weight (Neumark-Sztainer, 2005). Experimental studies have shown that even in the absence of attractiveness- and thinness-related cues of competitors, intrasexual status motives are able to trigger eating attitudes that resemble eating disorders in young women (Li et al., 2010; Castellini et al., 2017). A similar effect is not seen in heterosexual men (Li et al., 2010). Furthermore, eating disorders are much more common among homosexual men than in heterosexual men (Li et al., 2010; Calzo et al., 2018). A potential explanation for these findings is that intrasexual competition in homosexual men is focused on physical attractiveness, because homosexual men know that signals of youth and physical attractiveness are important mate preferences for other homosexual men (Li et al., 2010). Homosexual men also respond to intrasexual status competition with negative eating attitudes and worse perceptions about their own body image (Li et al., 2010).

If intrasexual competition is a significant factor in the development of eating disorders, individuals who are especially oriented toward the attainment of mating-related social status would have a higher risk of having eating disorders. Indeed, eating disorders are triggered most often around the age when intrasexual competition is strongest (Li et al., 2010). In addition, it has been shown that girls at schools with high proportions of female students have an elevated probability of developing eating disorders (Bould et al., 2016), which suggests that a higher intrasexual competitive environment increases the prevalence of eating disorders (cf. Baumeister et al., 2017; Saunders and Eaton, 2018).

Eating disorders are often socially contagious in friendship groups and may spread in the school environment (Bould et al., 2016). For example, if one’s friends have a low BMI due to eating disorder(s), one might perceive one’s own body to be relatively large, leading to higher body dissatisfaction and a higher probability of developing eating disorders. This sociodevelopmental etiology of eating disorders is supported by findings that in schools with greater proportions of underweight girls, other girls are more likely to try to lose body weight (Mueller et al., 2010).

There are also other hypotheses that have received less empirical support, such as (3) the *reproductive suppression hypothesis*, which suggests that AN is an adaptive attempt at reproductive suppression by the affected women (Wasser and Barash, 1983; Surbey, 1987; Voland and Voland, 1989). (4) The *parental manipulation hypothesis* suggests that AN is maintained by kin selection: parents manipulate female offspring to facilitate a strategic shift in reproductive investments between siblings (Voland and Voland, 1989). (5) The *reproductive suppression by dominant females hypothesis* posits that AN is a manifestation of reproductive suppression of subordinate females by dominant females during the process of female-female reproductive competition (Mealey, 2000). (6) The *adapted to flee famine hypothesis* suggests that symptoms of AN (such as hyperactivity and restriction of eating) enabled migration during famines to reach areas with more abundant food (Guisinger, 2003).

These hypotheses are focused on AN, leaving other eating disorders, especially BED, without an explanation. Hypotheses 3–5 completely fail to explain why eating disorders occur in men as well. They also fail to explain why sexual orientation influences the probability of having eating disorders (Li et al., 2010; Calzo et al., 2018). Existing research provides no support for the idea that individuals with anorexia nervosa would be socially subordinate individuals who adopt a “losing strategy” (Faer et al., 2005). Most of the hypotheses listed above are based on the idea that eating disorders are evolutionary adaptations.

In contrast to the other hypotheses, the intrasexual competition hypothesis does not suppose that eating disorders are adaptations. Furthermore, it does not apply only to AN, but it sees the whole spectrum of eating disorders as a pathological consequence of a mismatch between women’s adaptations for intrasexual competition and the modern environment in which those adaptations go awry. Despite the explanatory power of the intrasexual competition hypothesis, prior work on the hypothesis has been insufficiently formulated to give a full account of the evolutionary origins of eating disorders. We therefore extend it with the *mismatch hypothesis of eating disorders*.

### The Mismatch Hypothesis of Eating Disorders

Large-scale obesity is an evolutionary novelty. Human cultural evolution has led into a situation in which large quantities of *energetically dense* and *gustatorily hyper-rewarding* foods are readily available for most individuals in developed countries (Lindeberg, 2010; Power, 2012; Rozin and Todd, 2015; Corbett et al., 2018). Extracting energy from the environment does not entail a substantial energetic cost for most modern humans living in developed societies. The current energetic abundance that modern developed populations enjoy is an evolutionary novelty: ancestral humans were forced (on average) to expend higher amounts of energy to acquire food resources than modern humans are. This simple energetic disequilibrium (calories in > calories out) has led to an obesity epidemic and a swathe of modern health problems (Lindeberg, 2010; Power, 2012; Corbett et al., 2018), including with mental health (Milaneschi et al., 2018; Rantala et al., 2018).

Humans have a sophisticated suite of evolved psychological mechanisms (modules) responsible for food intake (King, 2013; Al-Shawaf, 2016; Rolls, 2017; Love and Sulikowski, 2018) and another suite of mechanisms (modules) responsible for mating (Weekes-Shackelford and Shackelford, 2014; Luoto, 2019a, b). The current environments of relative energy abundance (Lindeberg, 2010; Power, 2012) have created an evolutionarily novel conflict between psychological modules responsible for food intake and mating. On the one hand, humans are evolved to take full advantage of the presence of food supplies (Chakravarthy and Booth, 2004; King, 2013; Al-Shawaf, 2016); on the other, humans are evolved to signal their reproductive potential via phenotypic sexual ornaments (Sugiyama, 2015; Lassek and Gaulin, 2019). Cultural evolution has for the first time in human evolutionary history created a situation in which these psychological adaptations are in large-scale contradiction with one another. Thus, the *mismatch hypothesis of eating disorders* recognizes the novel situation in which previously co-adapted psychological mechanisms of food intake and mating become antagonistic. This antagonism creates a situation in which an individual is torn between opposing incentives: food rewards and mating rewards. The simultaneous presentation of conflicting adaptive problems constitutes an *adaptive metaproblem* (Al-Shawaf, 2016). The fundamental antagonism that the abundance of calorically dense and sensorily rewarding food (Lindeberg, 2010; Rozin and Todd, 2015) has caused between mating motives and food rewards drives one such adaptive metaproblem in contemporary humans, manifesting ultimately in various eating disorders.

The mismatch hypothesis could be falsified by showing that eating disorders are equally prevalent in traditional hunter-gatherer societies as they are in modern developed societies. Hunter-gatherer subsistence styles are somewhat comparable to the conditions of the human environment of evolutionary adaptedness (e.g., Al-Shawaf, 2016; Lewis et al., 2017). We are not aware of any evidence on the existence of AN, BN and BED in hunter-gatherer societies – on the contrary, hunger seems to be a pervasive aspect of modern hunter-gatherer societies (reviewed in Al-Shawaf, 2016).

The mismatch hypothesis is indirectly supported by non-human animal research which has shown that obesity becomes a significant problem only when humans keep animals in captivity (Power, 2012). Captivity represents an evolutionarily analogous condition for non-human animals as modern sedentary lifestyles do for humans (Williams, 2019), leading to a substantially increased prevalence of obese phenotypes under both circumstances (Power, 2012). These findings highlight the utility of framing eating disorders in the context of the evolutionary mismatch hypothesis, which we propose as a necessary extension to the intrasexual competition hypothesis. Besides eating disorders, the mismatch hypothesis also explains modern epidemics of several non-communicable diseases, such as type 2 diabetes, coronary artery disease (Corbett et al., 2018) and many other mental health problems (Li et al., 2018; Rantala et al., 2018).

## A Psychoneuroimmunological Model of Eating Disorders

Intrasexual competition for thinness and the adaptive metaproblem that arises from the abundance of sensorily rewarding and calorically dense foods (King, 2013; Rozin and Todd, 2015) seem to provide a plausible ultimate explanation for the pursuit of thinness in women living in developed societies. However, these hypotheses do not explain why only a fraction of women and homosexual men develop eating disorders. In addition, the hypotheses do not explain why some people develop such a strong obsession to lose weight that they starve themselves to death, while others binge eat and become overweight. The hypotheses also fail to explain the existence of non-fat-phobic AN (see section “Autoimmunity and Eating Disorders”).

Scientific progress depends on a good fit between theory and empirical evidence (Mathot and Frankenhuis, 2018). This fit is currently lacking between theory from evolutionary psychiatry and clinical evidence on eating disorders. We therefore posit the existence of proximate mechanisms that explain between-individual and within-individual variation in eating disorders, further improving the fit between theory and empirical findings. We propose a new model that explains the findings that (1) intensified intrasexual competition leads to eating disorders in only a small proportion of women; (2) this subset of women is likely to develop different eating disorders that entail the opposite phenotypic outcomes of extreme thinness and obesity; and (3) patient diagnoses may shift between eating disorders over time.

### Eating Disorders and Obsessive Compulsive Disorder

According to diagnostic criteria, obsession with physical exercise, appearance and food are common in eating disorders (American Psychiatric Association, 2013). These obsessions lead to emotional discomfort and to the development of series of behaviors like checking weight, exercising, purging or fasting. In addition to these classical symptoms of eating disorders, many other obsessive-compulsive traits, like doubting, checking and the need for symmetry and exactness are much more common in BN and AN patients than in psychiatric control groups (Cassidy et al., 1999). Some patients with eating disorders have visual or tactile checking rituals, such as touching body parts repetitively or viewing one’s body shape in the mirror (Legenbauer et al., 2014). Thus, the behaviors of eating disorder patients have many similarities with OCD behaviors (Bastiani et al., 1996; Garcia-Soriano et al., 2014). In a Swedish multigenerational family and twin study that included 19,814 participants with a diagnosis of OCD and 8,462 with AN (6.4% males), it was found that women with OCD had a 16-fold diagnosis of AN, whereas males with OCD had a 37-fold increased risk (Cederlof et al., 2015). AN and BN are also associated with personality traits linked to OCD, such as perfectionism and neuroticism (Cassidy et al., 1999; Anderluh et al., 2003; Halmi et al., 2005; Altman and Shankman, 2009). In addition, AN is more common in unaffected relatives of individuals with OCD, compared to the relatives of matched controls, suggesting shared genetic risk factors (Kaye et al., 1993). Accordingly, a GWAS meta-analysis found a genetic correlation between AN and OCD phenotypes (Anttila et al., 2018).

A recent positron emission tomography (PET) study found neuroinflammation in OCD patients; in particular, they have elevated microglia activity in their brains (Attwells et al., 2017). The distress associated with preventing compulsive behaviors is strongly correlated with neuroinflammation in the orbitofrontal cortex (Attwells et al., 2017). It is likely that neuroinflammation causes a cascade of biochemical events culminating in a dysregulation of neurohormones, neuropeptides and neurotransmitters which causes OCD symptoms. However, prior research (Attwells et al., 2017) has not been able to explain why OCD patients have neuroinflammation.

Although the obsessions in OCD cause significant stress for patients, stress itself seems to play an important role also in the onset of OCD (Toro et al., 1992; Behl et al., 2010; Adams et al., 2018). Stress triggers OCD symptoms and increases their frequency and severity (Findley et al., 2003). Experimental studies in non-human animals have shown that stress increases neuroinflammation and elevates microglia activity (reviewed in Calcia et al., 2016). Thus, chronic stress might be a source of the neuroinflammation that occurs in the OCD phenotype. Since stress and the activation of the HPA axis have such an important role in OCD (Sousa-Lima et al., 2019), one could expect a similar association with stress and eating disorder symptoms.

### Eating Disorders and Stress

Individuals with BN and AN are trying to lose weight to meet “the beauty ideal” and to persist in intrasexual competition for thinness (cf. Abed, 1998). AN and BN patients respond to competition by high stress hormone levels which become chronic over time (see Soukup et al., 1990; Rojo et al., 2006). Stress can be caused by peer and societal pressures to have the “perfect body type” (Castellini et al., 2017), while feelings of shame and guilt about one’s self image can cause individuals to continue in a vicious cycle of stress. Some patients with AN have identified retrospectively that negative comments about their body weight have been the triggering event for AN (Dignon et al., 2006). Especially in sports where low body weight is a competitive factor, requirements for thinness may trigger an eating disorder (Joy et al., 2016; Arthur-Cameselle et al., 2017). The same is true in the fashion world, dance and ballet (Marquez, 2008). The requirement to lose weight in sport and fashion may cause body dissatisfaction and social stress (cf. Castellini et al., 2017).

Retrospective research on patients with AN and BN has identified six other triggering events for eating disorders: (1) school transitions, (2) death of a family member, (3) relationship changes, (4) home and job transitions, (5) illness/hospitalization and (6) abuse, sexual assault or incest (Berge et al., 2012). Common to all of these triggering events is that they are known to increase stress. DSM-5 therefore states that AN onset is often associated with stressful life events (American Psychiatric Association, 2013).

Chronic stress is known to upregulate the immune system (reviewed in Stanton et al., 2018; Rohleder, 2019). Studies in humans and other animals have shown that social stressors are particularly potent triggers of the production of proinflammatory cytokines that may promote low-grade peripheral inflammation and neuroinflammation. Social rejection in humans is associated with increased levels of tumor necrosis factor-α (TNF-α) and interleukin-6 (IL-6) (Slavich et al., 2010). Accordingly, a meta-analysis that included 23 studies found that AN patients have significantly increased levels of TNF-α and IL-6, suggesting that AN patients have an upregulated immune system (Dalton et al., 2018). However, studies have not been able to exclude the possibility that increased IL-6 occurs because of weight loss. Since IL-6 stimulates lipolysis (Wedell-Neergaard et al., 2019), it is not clear whether increased levels of IL-6 are caused by malnutrition or inflammation, or both. Nevertheless, Dalton et al. (2018) found that patients with AN also have elevated levels of IL-15. IL-15 is associated with neuroinflammation (Pan et al., 2013), suggesting a link between AN and neuroinflammation.

As with AN patients (Solmi et al., 2015; Dalton et al., 2018), individuals with OCD also have increased levels of TNF-α and IL-6 (Konuk et al., 2007). Since AN and BN patients are often diagnosed with OCD (and since losing weight becomes a strong obsession for them), we hypothesize that neuroinflammation triggered by chronic stress underlies AN and BN – as it does with OCD (cf. Attwells et al., 2017). Indirect evidence for this hypothesis comes from observations that 74% of patients with AN and BN suffer from migraine (Brewerton and George, 1993; Brewerton et al., 1993; D’Andrea et al., 2009), which is a neuroinflammatory disease (Malhotra, 2016). Since starvation is known to increase stress hormone levels (Naisbitt and Davies, 2017), it appears that self-induced starvation may strengthen the stress-induced obsession to lose weight in AN patients. This feedback loop can create a vicious cycle which can be difficult to stop and which can escalate up to life-threatening levels. Interestingly, the stress hormone cortisol that is upregulated in AN patients (see Soukup et al., 1990; Rojo et al., 2006) is one of the hormones that increases gluconeogenesis in humans. Gluconeogenesis is the process of synthesizing glucose in the body from protein or fat, to be used as energy by the body (Khani and Tayek, 2001). By increasing stress hormone levels, gluconeogenesis can increase neuroinflammation during dieting and starvation in AN patients.

Experimental studies in humans and other animals have shown that proinflammatory cytokines reduce appetite and may cause sickness-induced anorexia (Dantzer, 2009). The mesolimbic reward system, which processes appetitive motivation and hedonic value of food, does not work as effectively in AN patients as in healthy controls or those with other eating disorders (Ceccarini et al., 2016). Thus, eating may not constitute an equally hedonic experience for AN patients as it does for unaffected individuals (cf. Stanton et al., 2018). This hedonic decline may partly contribute to the efficiency of AN patients’ dieting, while most healthy dieters fail in their efforts (see Mann et al., 2007).

### Binge Eating and Stress

As with BN and AN, we hypothesize that in many cases, also BED is triggered by intrasexual competition for thinness. Indirect evidence for this hypothesis is given by findings showing that BED patients have low self-esteem, general body dissatisfaction (Pearl et al., 2014) and elevated psychological distress (Castellini et al., 2017; Mustelin et al., 2017). They are prone to overestimate their weight and to see their body shape in a negative light (Pearl et al., 2014). Despite weight loss intentions, BED patients end up binge eating and gaining more weight, often leading to obesity. Castellini et al. (2017) reported that binge eating was associated with dysfunctional body image esteem and greater sexual distress in a non-clinical population of women, further supporting our hypothesis that BED is triggered by intrasexual competition for thinness. This hypothesis could be challenged by showing that BED symptoms are an effect of BED rather than its cause. However, a study that compared normal-weight BED individuals and obese BED individuals found that the normal-weight ones had a stronger urge to lose weight than the obese ones (Goldschmidt et al., 2011). There were no between-group differences in overvaluation of shape or weight, suggesting that these symptoms are not caused by comorbid obesity (Goldschmidt et al., 2011).

While many people lose their appetite when feeling heavily stressed, even a mild psychological stressor or a negative affective episode may trigger binge eating in BED or BN patients (Masheb et al., 2011). Usually stress activates the sympathetic nervous system and the body’s fight-or-flight response. Under these circumstances, corticotropin-releasing factor (CRF) suppresses appetite by affecting the digestive system and decreasing the sense of hunger. This is why individuals with BED do not experience binge cravings and binge eating during the acute stress, but in the privacy of their homes and when alone long after the acute stressor has subsided (Masheb et al., 2011). Binge eating can be seen as a way to “escape” from a negative aversive emotional state (Burton and Abbott, 2019).

BED and BN patients have an express wish to lose weight, and therefore it is important to understand why it is so difficult for them to resist binge eating. The reason might lie in their dieting practice. In calorie-restricted rats, psychological stress has been shown to trigger binge eating episodes if subjects have an opportunity to eat food items that are heavy in sugar and fat (Hagan et al., 2002, 2003). Likewise, footshock stress with calorie restriction leads rats to consume twice the normal amount of food (Boggiano et al., 2005). Food-restricted rats that are experimentally stressed develop inflammation in discrete brain regions which directly or indirectly regulate food intake; these rats also develop binge-like eating behaviors (Alboni et al., 2017). Correspondingly in human subjects, psychological stress may trigger binge eating in healthy dieters if highly palatable food is available (Oliver and Wardle, 1999; see also Castellini et al., 2017; Klatzkin et al., 2018).

An evaluation of the psychophysiological state of patients provides further insight into BED. BED patients have higher stress responsivity than controls (Klatzkin et al., 2018). BED patients may turn to binge eating more easily than controls precisely because of their elevated stress responsivity (cf. Klatzkin et al., 2018). One reason for their high stress responsivity may be inflammation caused by visceral fat tissue (Shields et al., 2017; Krams et al., 2018; Rohleder, 2019). Although psychosocial stressors are present in the lives of most people, self-regulatory abilities buffer individuals against negative health outcomes that are frequently caused by stress (Evans and Fuller-Rowell, 2013; Shields et al., 2017). Accumulating evidence suggests, however, that inflammation may cause widespread biobehavioral alterations that promote self-regulatory failure (Shields et al., 2017). BED patients have 88% higher sensitive CRP values than controls matched for body weight, suggesting that BED patients have severe inflammation in their body (Succurro et al., 2015). The peripheral inflammation may therefore reduce self-regulatory capacity (Shields et al., 2017) in BED patients and further increase their stress responsivity. This is because proinflammatory cytokines produced by immune cells or adipocytes are known to stimulate the HPA axis (Yau and Potenza, 2013). This mechanistic link may cause a vicious cycle, leading to obesity (cf. Shields et al., 2017; Milaneschi et al., 2018) and, as we suggest, to BED. Peripheral inflammation is a potential causal mechanism that explains why mood disorders are so common among BED patients: inflammation, for instance, increases the likelihood that an adaptive mood change turns to maladaptive clinical depression (cf. Luoto et al., 2018; Rantala et al., 2018). Genetic factors may further increase the comorbidity between these disorders (as reviewed in section “Risk Factors for Eating Disorders”).

### The Neurochemistry of Anorexia Nervosa and Bulimia Nervosa

Serotonin (5-hydroxytryptophan) is known to influence impulse control, obsessionality, mood and appetite (Bailer and Kaye, 2011; Dalley and Roiser, 2012; Garcia-Garcia et al., 2017). Treatments that upregulate serotonergic activity tend to reduce food consumption, while treatments that downregulate serotonergic activity increase food consumption and promote weight gain (reviewed in Bailer and Kaye, 2011; see also Alonso-Pedrero et al., 2019). Studies on AN patients have reported serotonergic system dysfunction (reviewed in Bailer and Kaye, 2011; Riva, 2016). Interestingly, IL-15 is upregulated in AN patients (Dalton et al., 2018), and studies on mice have shown that IL-15 upregulates the serotonergic system (Wu et al., 2011; Pan et al., 2013).

In the acute phase of AN (when individuals are underweight), patients have significantly lower levels of serotonin metabolites in their cerebrospinal fluid than healthy controls (Kaye et al., 1984, 1988). They also have blunted prolactin response to drugs with serotonin activity and reduced ^3^H-imipramine binding, further suggesting reduced serotonergic activity (Bailer and Kaye, 2011). Since serotonin is synthesized from an amino acid called tryptophan, an essential amino acid that must be obtained from food, the most plausible explanation for low serotonin metabolism in AN patients during the acute phase of the illness is that it results from starvation/dieting (Kaye et al., 2009; Haleem, 2012). In contrast, individuals who have recovered from AN have elevated serotonin levels (Kaye et al., 1991). An experimental study found that a reduction of dietary tryptophan reduced anxiety and elevated mood in women with AN, but had no effect on control women (Kaye et al., 2003).

**FIGURE 1:**
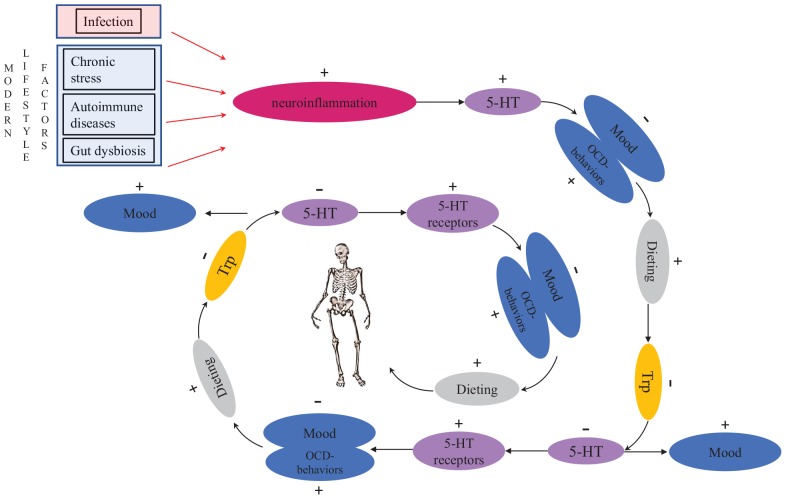
The vicious, potentially fatal serotonergic-homeostatic stress/starvation cycle that leads to anorexia nervosa. TRP, tryptophan; 5-HT, serotonin.

AN patients are known to have high levels of anxiety, obsessionality and harm avoidance both premorbidly and after recovery. They may also have higher levels of serotonin premorbidly, resulting in a dysphoric state (Bailer and Kaye, 2011). Kaye et al. (2009) suggested that dieting/starvation makes AN patients feel better by decreasing serotonergic activity in the brain. These individuals may also get positive feedback from their peers about their thinner appearance, which further motivates them to continue starvation. As a result of tryptophan depletion caused by starvation, the brain responds by increasing the number of serotonin receptors to utilize the remaining serotonin more efficiently (Kaye et al., 2009). This leads to a vicious homeostatic cycle (Figure 1), because in order to feel better, AN patients need to reduce tryptophan even more, leading to reduced food consumption (Kaye et al., 2009). If the patient starts to eat food that has tryptophan in it, serotonin levels arise sharply which causes extreme anxiety and emotional chaos (Kaye et al., 2009). This makes the recovery of AN patients so difficult (Kaye et al., 2009). The drop of serotonin levels during the acute phase of the illness due to shortage of tryptophan (Riva, 2016) may explain the serious body image disturbances that are typical in AN. Although the exact neurophysiological mechanism that causes such disturbances is not known, the mechanism is probably similar to the low self-esteem often seen in depression (cf. Orth and Robins, 2013).

Kaye et al. (2009) hypothesized that individuals with AN have an intrinsic defect in their serotonergic system and that gonadal steroid changes during menarche or stress-related issues on adolescent individuation might further alter activity of the serotonergic system. However, this explanation is not able to account for increased AN prevalence in modern societies nor for the occurrence of eating disorders in men. *To link proximate mechanisms with ultimate causes*, it is important to consider the timing of the onset of AN in menarche and early adulthood as potentially caused by intensified intrasexual competition for thinness around this central period in reproductive development. Intrasexual competition may cause chronic stress for individuals who are highly competitive (cf. Vaillancourt, 2013). Importantly, chronic stress is known to increase serotonin levels in brains both in humans (reviewed in Hale et al., 2012) and in other animals (e.g., Adell et al., 1988; Keeney et al., 2006; Vindas et al., 2016). Experimental studies in rats have shown that fasting reduces serotonin levels in brains (Haleem and Haider, 1996). The same mechanism can also occur in stressed people with an upregulated serotonergic system when they start dieting to alleviate the dysphoric state. In the aggregate, these findings explain one part of the mechanistic link between chronic stress and disordered eating (Figure 1).

There is also evidence on dysfunction in the serotonergic system in bulimia nervosa (reviewed in Sjögren, 2017), but in a different way than in AN. The serotonin levels of BN patients drop more than in healthy controls even during short periods of fasting (e.g., during sleep), leading to mood irritability and binge eating episodes (Steiger et al., 2001). These abnormalities in the functioning of the serotonergic system persist after recovery, suggesting that they might have existed already before the onset of BN (Kaye et al., 2001). In contrast to AN patients, tryptophan depletion in BN patients lowers mood and causes an urge to binge eat (reviewed in Sjögren, 2017). A neuroimaging study found increased 5-HT1A binding in BN patients compared to healthy controls (Galusca et al., 2014). Activation of the serotonin receptor 5-HT_2__C_R reduced binge eating of palatable food in a rat model (Martin et al., 1998; Fletcher et al., 2010; Higgins et al., 2013; Price et al., 2018). Likewise, SSRI medication reduced the urge to binge eat (reviewed in Tortorella et al., 2014). These findings support the hypothesis that BN patients have reduced serotonin production in the central nervous system.

### Sources of Individual Differences in Responses to Intrasexual Competition

**FIGURE 2:**
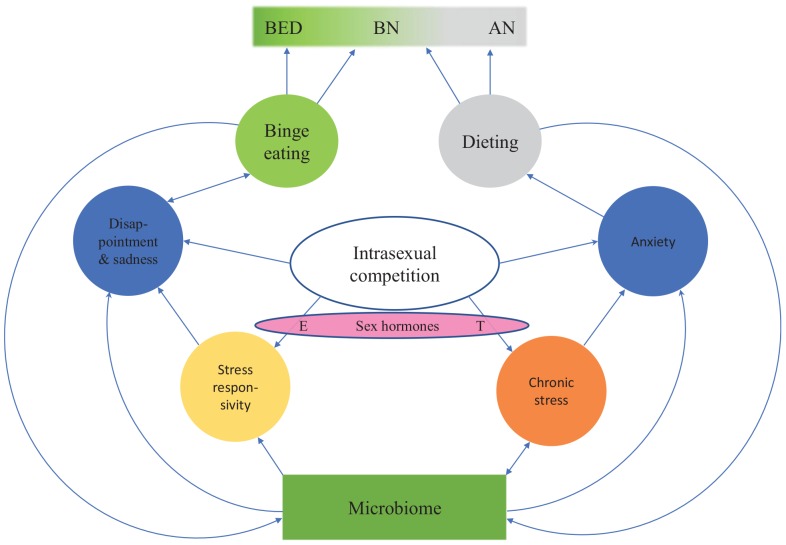
An evolutionary psychoneuroimmunological continuum model for eating disorders. The model shows how intrasexual competition for thinness leads to various emotion states (blue circles). Individual differences in these emotion states and the resulting eating behaviors (green and gray circles) are partially driven by individual differences in prenatal sex hormone exposure as well as premorbid and current microbiome constitution. Thus, between-individual and within-individual variation in eating disorders can arise from dynamic shifts in inflammation, stress, serotonin levels, tryptophan concentrations and the microbiota. BED, binge eating disorder; BN, bulimia nervosa; AN, anorexia nervosa; E, estrogen; T, testosterone.

There are major individual differences in responses to female-female intrasexual competition: some individuals binge eat while others starve to death (Figure 2). Most young women are exposed to intrasexual competition for thinness, but only a small proportion of them develop an eating disorder. The reason for this variation seems to be associated with individual differences in stress responsivity and in the functioning of the serotonergic system. To understand the etiology of eating disorders, one central question is: what are the primary sources of heightened stress responsivity and dysfunction of the serotonergic system in people with eating disorders?

A potential factor driving these individual differences might be gut dysbiosis (cf. Temko et al., 2017). A substantial amount of empirical evidence in other animals suggests that gut microbiota influences stress responsivity, anxiety-like behavior and the set point for activation of the neuroendocrine hypothalamic-pituitary-adrenal (HPA) stress axis (reviewed in Foster et al., 2017; see also Molina-Torres et al., 2019). Changes in stress-related physiology and behavior modulated by gut dysbiosis may result from the alteration of microbial composition by antibiotic exposure, poor diet, lack of breastfeeding, birth by Cesarean section, infection, stress exposure and many other environmental factors (reviewed in Foster et al., 2017).

Important evidence for the hypothesized mechanism between eating disorders and the microbiota is provided by the finding that 64% of individuals with eating disorders have been diagnosed with irritable bowel syndrome (Perkins et al., 2005). Recent studies have found that AN patients deviate from controls in the abundance, diversity and microbial composition of the fecal microbiota (reviewed in Schwensen et al., 2018), which remain significantly different from those of healthy controls even after refeeding (Kleiman et al., 2015; Mack et al., 2016). Although some of the deviations in microbiota may be caused by dieting – with a limited diet limiting microbial diversity (as found in studies on insects as well as humans: Krams et al., 2017; Stevens et al., 2019) – it is also possible that these individuals have had deviant microbiota premorbidly. Recent studies have shown that stress disturbs gut microbiota (Gao et al., 2018; Partrick et al., 2018; Molina-Torres et al., 2019). Thus, chronic stress that has triggered an eating disorder may have changed microbiota in patients with eating disorders (cf. Seitz et al., 2019). Unfortunately, studies on the microbial composition of patients with BED and BN are currently lacking. Studies testing premorbid microbiota of eating disorder patients would be particularly valuable. Interestingly, a large Swedish study (Hedman et al., 2019) found that risk of celiac disease was increased by 217% within the first year after AN diagnosis. Likewise, Crohn’s disease was three times more common in AN and ulcerative colitis was 2.3 times more common in AN than in controls (Wotton et al., 2016). Since these diseases are triggered by dysbiosis (e.g., Ni et al., 2017), their increased prevalence in AN patients provides additional support for the link between dysbiosis and AN.

Studies in germ-free mice have shown that early-life microbiota absence leads to increased tryptophan concentration in plasma and increased serotonergic activity in the brain (Clarke et al., 2013). These changes can be normalized by providing mice with probiotic bacteria that are known to influence tryptophan metabolism (Clarke et al., 2013). In the aggregate, the studies reviewed above indicate that abnormalities in serotonergic activity in eating disorders may be at least partly caused by deviations in gut microbiota. We believe that this is an important avenue for future studies (cf. Seitz et al., 2019), although a significant amount of work is still required before we will be in a position to develop microbiome-based treatments for eating disorders.

### Eating Disorders as a Continuum

It is an important but theoretically and clinically underappreciated finding that more than 50% of women diagnosed with AN develop BN (Bulik et al., 1997). One reason why AN changes to BN might be that as an individual’s nutritional status improves, their gut microbiota changes, which may in turn influence their stress responsivity and the functioning of the serotonergic system. This hypothesis is supported by findings showing that bulimic AN subtypes (AN-B) differ from restricting-type AN (AN-R) in microbial community composition (Mack et al., 2016), while refeeding changes the gut microbiota (Kleiman et al., 2015). In addition, after refeeding has increased tryptophan concentrations, homeostatic responses in the serotonergic system may lead with time to a state where serotonin levels undergo an excessive decrease, which in turn causes the urge to binge eat (cf. Steiger et al., 2001).

Interestingly, while a meta-analysis found that AN patients have increased cytokine concentration in plasma (especially IL-6 and TNF-α), cytokine levels in BN patients did not differ from controls (Dalton et al., 2018). Experimental studies in non-human animals have found that administration of IL-6 cytokines increases serotonin levels and reduces dopamine levels in nuclear accumbens, an effect amplified by stress (Song et al., 1999). Based on these findings, we hypothesize that if cytokine levels drop in AN-R patients, it may cause a reduction in serotonin levels to the extent that an urge to binge eat emerges, thus changing the eating disorder diagnosis of the patient to AN-B. This hypothesis is supported by the finding that AN-B patients have a lower level of inflammation than AN-R patients (Solmi et al., 2015).

Overall, these hypothesized shared mechanisms underlying eating disorders indicate that eating disorders are not separate disorders. Instead, they seem to form a continuum, with BED at one end of the spectrum and AN-R at the other end. BN and bulimic-type AN (AN-B) are located between the extremes (Figure 2). Previously, the transdiagnostic view of eating disorders was questioned (Birmingham et al., 2009), partially because the role of stress, neuroinflammation and gut dysbiosis in the etiology of eating disorders was not understood. The model presented in this article (Figures 1, 2) suggests that dynamic shifts in inflammation, stress, serotonin levels, tryptophan concentrations and the microbiota cause shifts in disordered eating behaviors. When the proximate mechanisms discussed above are integrated into a continuum model of eating disorders, we are in a significantly better position to explain why a person’s symptoms often change in the course of an eating disorder and why patients may subsequently be diagnosed with another eating disorder.

### Autoimmunity and Eating Disorders

All cases of eating orders are not necessarily caused by intrasexual competition for thinness. For example, there are AN patients without an intense fear of gaining weight or becoming fat. DSM-V (American Psychiatric Association, 2013) introduced the diagnosis ARFID (Avoidant/Restrictive Food Intake Disorder) to describe underweight patients who do not experience body image disturbance (i.e. non-fat-phobic AN). ARFID appears to be more common in developing countries than fat-phobic AN, which is significantly more common in developed countries (reviewed in Becker et al., 2006). Likewise, although AN and other eating disorders are much more common among homosexual than heterosexual men (Li et al., 2010; Calzo et al., 2018), eating disorders do occur in some heterosexual men. This may be difficult to explain only through intrasexual competition for thinness because thinness is not as important a mate preference for women as it is for men (Li et al., 2010).

If neuroinflammation plays an important role in AN, the crucial question is what causes neuroinflammation in these cases? Naturally, chronic stress that results in neuroinflammation may be caused by other factors than intrasexual competition. This can be true particularly in non-fat-phobic AN patients. Besides chronic stress, neuroinflammation may also be caused by autoimmune and autoinflammatory diseases (Najjar et al., 2013). Correspondingly, there are many case reports in which AN has been triggered by infections: these cases have been described as “autoimmune anorexia nervosa” (Sokol and Gray, 1997; Sokol, 2000). In some cases, OCD has been observed to follow infection (American Psychiatric Association, 2013), providing more support for the association between activation of the immune system and the onset of OCD and AN (Figure 1). Just like fat-phobic AN patients, non-fat-phobic AN patients may learn to alleviate anxiety by dieting. This can lead to the same vicious stress/starvation cycle as in fat-phobic AN (Figure 1). Furthermore, our psychoneuroimmunological model provides an explanation also for those historical cases of non-fat-phobic anorexia nervosa that existed in historical societies that did not have a thin beauty ideal (cf. Arnold, 2013).

In a large nation-wide population study conducted in Denmark, Zerwas et al. (2017) found that autoinflammatory or autoimmune diseases increase risk for AN by 36%, BN risk by 73% and eating disorder not otherwise specified (EDNOS) risk by 72%. This effect was stronger for boys than for girls (Zerwas et al., 2017). For boys, having any autoinflammation increased risk for EDNOS by 740%. A large-scale Swedish study reported that any preceding autoimmune diseases increased AN risk by 59% (Hedman et al., 2019). Another large study analyzing the genetic connection between eating disorders and autoimmune diseases did not identify any genetic overlap between anorexia nervosa and autoimmune diseases (Tylee et al., 2018). This suggests that environmental rather than genetic factors cause the association between AN and autoimmune diseases.

We interpret these findings as providing broad support for our psychoneuroimmunological model for eating disorders for four reasons: (1) chronic stress is known to cause autoimmune diseases (Song et al., 2018), (2) many autoinflammatory and autoimmune diseases are known to increase neuroinflammation (Najjar et al., 2013), (3) activation of the immune system is known to increase stress responsivity (Yau and Potenza, 2013), and (4) dysbiosis in gut microbiota may lead to the onset of autoinflammatory diseases (Lukens et al., 2014). As reviewed in section “The Neurochemistry of Anorexia Nervosa and Bulimia Nervosa”, dysbiosis is also common in eating disorders.

Thus, in the case of ARFID (non-fat-phobic AN), individuals with neuroinflammation may learn that dieting and fasting can alleviate anxiety and dysphoria because dieting and fasting reduce autoimmune responses (cf. Hafstrom et al., 1988) and downregulate the serotonergic system (cf. Kaye et al., 2009), leading to a vicious dieting cycle and eventually to AN. This psychoneuroimmunological mechanism may explain why heterosexual and asexual men sometimes suffer from AN (cf. Carlat et al., 1997) even when strong intrasexual competition for thinness is not present to the same degree as it is in heterosexual women (cf. Abed et al., 2012).

## Comorbidity of Eating Disorders

Eating disorders typically have a high comorbidity rate with other mental disorders (Keski-Rahkonen and Mustelin, 2016). For example, 93–95% of adult AN patients had a comorbid mood disorder, 55–59% had anxiety disorder and 5–20% had a substance-related disorder (Blinder et al., 2006). Research on comorbidity of mental disorders in BN has shown that 94% of adult BN patients had a mood disorder, 55% had anxiety disorder and 34% had substance use disorder (Swanson et al., 2011). Comorbidities were less frequent for teenagers with BN: 49.9% had mood disorder, 66.2% had anxiety disorder, substance abuse occurred in 20.1% of BN teenagers while 57.8% had a behavioral disorder (Swanson et al., 2011).

Because BED has only recently been classified as a separate disorder, studies on comorbid mental disorders are scarce (cf. Olguin et al., 2017). A large epidemiological study on US teenagers found that 45.3% of individuals with BED had a comorbid mood disorder, 65.2% had anxiety disorder, substance abuse occurred in 26.8% of the teenagers while 42.6% had a behavioral disorder (Swanson et al., 2011).

OCD, which shares many similarities with eating disorders, has been associated with gut microbiome dysregulation (Turna et al., 2017) and altered serotonin activity in the brain (Lissemore et al., 2018). Just like eating disorders, OCD is associated with high comorbidity with other mental disorders (Hofmeijer-Sevink et al., 2013). Since gut microbiome dysregulation and chronic stress are both associated with mood disorder and anxiety disorder (reviewed in Bekhbat and Neigh, 2018; Liang et al., 2018), the most plausible explanation for the occurrence of these comorbidities is that in OCD and eating disorders, these comorbidities are caused by dysbiosis and heightened sensitivity to stress.

Importantly, many AN symptoms seem to be symptoms of *starvation*, not of a mental disorder. For example, in the famous Minnesota starvation study, 36 healthy men were subject to semi-starvation for 6 months (Keys, 1950). The men began subsequently to display symptoms similar to eating disorders, such as ritualistic eating, preoccupation with food and eating. Starved men also developed hoarding and obsessive collecting behaviors, suggesting that starvation may cause or reinforce symptoms resembling obsessive-compulsive disorder (OCD). Some starved men tended to read cookbooks, dream about food and constantly speak about it (Keys, 1950). A similar obsession with food is commonly observed in AN patients (Crisp, 1983).

The starved men also became irritable, anxious and depressed, which suggests that starvation led to starvation-induced depression (cf. Rantala et al., 2018). In many subjects, the symptoms persisted also after refeeding. Prolonged starvation episodes lead to apathy and social withdrawal (cf. Keys, 1950), which are also common AN symptoms. The Minnesota starvation study showed that as soon as the starvation experiment was over, many men expressed concerns about gaining too much weight and “becoming flabby” (Keys, 1950). Similar kind of examples can be found in case reports and diaries during famines (Keys, 1950). Despite being emaciated, most of the men did not see themselves as underweight (Keys, 1950). Thus, it seems possible that starvation may fuel the distorted self-image that is characteristic of AN and that starvation itself causes the kind of psychopathology seen in AN patients.

## Risk Factors for Eating Disorders

### Genetic Factors and Neuroinflammation

Family, twin and adoption studies have consistently demonstrated that genetic factors contribute to the variance in susceptibility to eating disorders. Heritability estimates for BED range between 41 and 57%; BN heritability estimates range between 30 and 83%, while AN has a heritability of 28–78% (Thornton et al., 2011). Eating disorders are familial: female relatives of individuals with AN are 11.3 times more likely to develop AN than relatives of individuals without AN; female relatives of individuals with BN are 12.3 more likely to develop BN than relatives of individuals without BN (Strober et al., 2000). Few specific genetic risk factors have been conclusively identified for eating disorders (reviewed in Mayhew et al., 2018), although a recent study indicated eight genetic loci underlying AN etiology, suggesting metabo-psychiatric origins for the disorder (Watson et al., 2019). Twin studies have revealed that there is shared transmission between eating disorders and anxiety disorders (Keel et al., 2005), between AN and OCD (Altman and Shankman, 2009) and between BN and panic disorder (Keel et al., 2005). The most probable explanation for these findings is that the shared transmission is caused by vulnerability to neuroinflammation and stress responsivity, as suggested by the evidence reviewed above. This vulnerability to neuroinflammation and stress may explain why GWAS studies have found genetic correlations between AN and many other mental disorders like schizophrenia, major depressive disorder, bipolar disorder and autism (Anttila et al., 2018; Sullivan et al., 2018), because neuroinflammation plays a role in all of them (Najjar et al., 2013). Together with differences in microbiome composition, these genetic susceptibilities to neuroinflammation may partly explain whether intrasexual competition leads to BED, BN or AN (cf. Figure 2).

### Childhood Maltreatment, Stress, Epigenetics and Microbiota

Childhood maltreatment in the form of sexual, emotional or physical abuse increases the risk for affective disorders (reviewed in Hoppen and Chalder, 2018). Childhood maltreatment increases the risk to develop an eating disorder by more than three times (Caslini et al., 2016). Childhood maltreatment increases stress responsivity in adulthood, an outcome partially driven by epigenetic mechanisms such as altered DNA methylation (DNAm) of the HPA axis genes (Bustamante et al., 2016). Chronic stress in childhood can also affect the microbiome in such a way that an altered, suboptimal microbiome predisposes individuals to increased stress (O’Mahony et al., 2016). In addition to increased sensitivity to stress, early-life stress can prime microglia, which may lead to a potentiated neuroinflammatory response to subsequent stress (reviewed in Calcia et al., 2016). Chronic stress has often been reported within the year prior to the onset of AN in epidemiological studies (Rojo et al., 2006). AN patients have reported higher levels of total lifetime stress and more difficulties coping with stress than healthy controls (Soukup et al., 1990). Retrospective studies have found that severe life stress differs between AN and control samples, predicting AN onset in 67% of cases (Schmidt et al., 2012).

### The Role of Sex Hormones

Prenatal and current sex hormone levels seem to partly influence whether intrasexual competition for thinness leads to eating disorders. Sex hormones also influence the type of eating disorder developed (Figure 2). Estrogen is known to stimulate HPA activity, thereby increasing stress responsivity (Kudielka and Kirschbaum, 2005). Androgens, in contrast, tend to reduce HPA activity and thereby reduce stress responsivity (Kudielka and Kirschbaum, 2005). Men usually show higher HPA activation in status-related situations whereas women show higher HPA activation in situations involving social rejection (reviewed in Del Giudice et al., 2011).

It could therefore be predicted that more masculine heterosexual women (Bártová et al., 2020), i.e., women with higher prenatal androgen exposure or higher current testosterone levels (Luoto et al., 2019a, b) – and, thus, a higher drive for social status (cf. Nave et al., 2018) – have a higher likelihood of developing AN. In contrast, more feminine women who are more sensitive to social rejection would be expected to have a higher risk of developing BED. Indeed, digit ratio (2D:4D, i.e., a biomarker of prenatal androgen exposure: Luoto et al., 2019a) is more masculine in AN patients than in BN patients, with controls having an intermediate digit ratio (Quinton et al., 2011). This suggests that AN patients might have experienced higher prenatal androgen exposure than BN patients and controls (for a detailed discussion of these developmental mechanisms in non-clinical populations, see Luoto et al., 2019a, b). In women, low prenatal testosterone levels and high pubertal ovarian hormone levels seem to increase risk of BED; in men, high prenatal testosterone levels seem to protect against BED (Klump et al., 2017). These findings highlight the role of sex hormones in phenotypic variation (Figure 2) and sex differences in eating disorders.

## Current Eating Disorder Treatment

The treatment of eating disorders is much less effective than the treatment of other mental disorders. Only 46% of AN patients recover completely, one third recover partly and in 20% AN remains as a chronic condition (Arcelus et al., 2011). The average duration of the illness is 6 years (Schmidt et al., 2016). Currently, there is no effective pharmacological treatment for AN. Selective serotonin reuptake inhibitors (SSRIs) are ineffective for AN (Davis and Attia, 2017). There is no approved medication to treat anorexia nervosa in the US or the EU (Bodell and Keel, 2010; Starr and Kreipe, 2014). AN treatment is therefore based on different kinds of therapies and efforts to restore weight (e.g., Brockmeyer et al., 2017; Harrison et al., 2018 and references therein). The efficacy of family-based treatment (FBT) is reportedly superior to other forms of psychotherapy (Starr and Kreipe, 2014).

The primary treatment for BN is cognitive behavioral therapy (CBT) that aims to change the negative thought patterns that underlie binge eating while also attempting to normalize eating behaviors (Fairburn, 2008). SSRIs slightly suppress binge eating behavior but do not typically eliminate it (Mitchell et al., 2013). The dropout rate in antidepressant treatment in BN patients is around 40% (Bacaltchuk and Hay, 2003). Current BN treatment is not particularly effective: follow-up studies have shown that in a 10-year period, only 50% of patients are fully recovered (Hay et al., 2009).

Antidepressants are only modestly effective against binge eating episodes in the short term, but their long-term efficacy is not known (McElroy et al., 2012). In addition, they do not help to reduce body weight and they do not appear to enhance the antibinge eating effects of CBT (McElroy et al., 2012). Second generation antipsychotics used in AN treatments in fact induce or exacerbate binge eating in patients with BED and BN (McElroy et al., 2012; Cuesto et al., 2017). Double-blind and placebo-controlled experiments have shown that an anti-epileptic agent, topiramate, is effective against binge eating episodes in BED with obesity. Topiramate has high anti-inflammatory properties, and has been shown to reduce neuroinflammation and oxidative stress in rats (Pinheiro et al., 2015). It has also been shown to attenuate stress-induced increased alcohol consumption in mice (Farook et al., 2009), suggesting that it may reduce stress responsivity. Interestingly, topiramate also effectively reduces compulsions in OCD (Rubio et al., 2006; Van Ameringen et al., 2006; Mowla et al., 2010; Berlin et al., 2011). Unfortunately, topiramate is not suitable as treatment for AN nor for patients who have a history of AN because topiramate reduces appetite and enhances weight loss as a side-effect. It may even induce AN in those with known risk factors for AN (Lebow et al., 2015). In the United States and Canada, the only approved drug for moderate and severe BED is lisdexamfetamine, which has also been used to treat attention deficit hyperactivity disorder (ADHD) (Heo and Duggan, 2017). Randomized double-blind trials have shown that lisdexamfetamine is more effective against binge eating than placebo. However, lisdexamfetamine has harmful side effects like dry mouth, headache and insomnia that lead to drug discontinuation in many patients (Heo and Duggan, 2017). In addition, it merely alleviates the symptoms rather than removing the underlying problem that causes the eating disorder, thus providing a suboptimal long-term solution (cf. Rantala et al., 2017).

## Eating Disorder Treatments Based on Psychoneuroimmunology

The psychoneuroimmunological model that we have presented has the potential to improve the effectiveness of eating disorder treatments. The model posits that, rather than providing family-based treatment and psychotherapy for AN patients, it might be more effective to try to reduce the obsession to lose weight by reducing neuroinflammation and chronic stress. We also suggest that CBT based on evolutionary psychiatry could further help shift patients’ self-image and attitudes of thin beauty ideals toward a healthier direction. Critically, our psychoneuroimmunological model suggests that lifestyle changes that reduce neuroinflammation and stress are expected to reduce AN symptoms, though further clinical studies are needed for this to be empirically verified.

### Medication for AN That May Target Neuroinflammation

Recent studies suggest that olanzapine (an atypical antipsychotic drug) treatment leads to significant weight gain in patients with AN (Dold et al., 2015; Himmerich et al., 2017). Studies in mice have shown that olanzapine reduces neuroinflammation (Sharon-Granit et al., 2016). It also suppresses TNF-α and IL-6 and increases IL-10 levels, which is an anti-inflammatory cytokine (Sugino et al., 2009). Thus, a possible mechanism of how olanzapine helps AN patients may be reduced neuroinflammation, though this remains to be empirically verified.

Case studies indicate a positive effect of anti-TNF-α treatment on AN (Solmi et al., 2013). Esalatmanesh et al. (2016) found that minocycline antibiotic that is known for its anti-inflammatory characteristics significantly reduced OCD symptoms in OCD-patients without causing any harmful side effects.

### Zinc, Anorexia Nervosa and Neuroinflammation

Evidence from clinical studies indicates that AN patients have low serum zinc levels and low rates of urinary zinc excretion (Katz et al., 1987). The severity of zinc deficiency is associated with the severity of AN, as well as with higher levels of depression and anxiety (Katz et al., 1987). Several randomized controlled trials of zinc supplementation have reported significant increases in subjects’ weight (Safai-Kutti and Kutti, 1986; Safai-Kutti, 1990; Birmingham et al., 1994; Birmingham and Gritzner, 2006). Zinc deficiency is known to be associated with an increased production of proinflammatory cytokines, especially TNF-α and IL-6 (Gammoh and Rink, 2017). Thus, we suggest that a possible mechanism between zinc supplementation and reductions in AN symptoms and weight gain may operate via reduced neuroinflammation. Reduced neuroinflammation, in turn, decreases obsessions. This intepretation is supported by findings from placebo-controlled trials which reported that attitudes toward eating and food became more positive especially in AN patients who ate zinc pills (in contrast with those who received placebo pills) (Khademian et al., 2014). Thus, it seems that zinc reduces OCD symptoms in AN patients. Interestingly, zinc supplements reduce symptoms also in patients with OCD who do not have eating disorders (Sayyah et al., 2012). Overall, more research is needed to understand the mechanism(s) through which zinc influences AN symptoms.

In addition to zinc, other micronutrients could also be used to reduce neuroinflammation. For example, AN patients have a deficiency of vitamin D (Veronese et al., 2015; Tasegian et al., 2016) and vitamin D supplementation is known to reduce inflammation (Grossmann et al., 2012; Zhang et al., 2012; Berk et al., 2013) as well as neuroinflammation (Koduah et al., 2017).

### Fecal Microbiota Transplantation and Probiotics

If scientific advances continue to provide empirical support for the role of the microbiota in the etiology of eating disorders, it is possible that fecal microbiota transplants from healthy individuals will be a part of future therapeutic treatments of eating disorders. The first published case study (de Clercq et al., 2019) reported significant body weight gain after fecal microbiota transplantation for a patient with AN. However, research in this field is in its infancy. In addition to fecal microbiota transplantation, gut microbiota might be therapeutically manipulated with probiotics or other supplements (Stevens et al., 2019). Unfortunately, although accumulating evidence suggests that probiotics are a promising adjuvant treatment to reduce inflammatory activation found in major depressive disorder (reviewed in Park et al., 2018), studies on the efficacy of probiotics as eating disorder treatments are currently lacking.

### Binge Eating Disorder (BED) Treatment Based on Evolutionary Psychiatry

BED treatment based on evolutionary psychiatry should, in our view, focus on stopping the vicious cycle of dieting efforts that fuel binge eating episodes. Patients should try to lose weight through healthy eating and exercising (cf. Lindeberg, 2010; Temko et al., 2017; Leone et al., 2018) rather than by trying to decrease calorie intake by fasting. This is a very similar approach to current CBT treatments of BED (cf. Hilbert, 2013). A healthy diet may also help to reduce BED patients’ stress responsivity: experimental studies in animals have shown that intestinal microbiota influences stress responsivity (Bravo et al., 2011). In addition, a healthy diet, especially omega-3 fatty acids and polyphenols, reduces inflammation (Ricordi et al., 2015), which is known to stimulate HPA-activity (Yau and Potenza, 2013; Rohleder, 2019) and deteriorate self-regulatory capacity (Shields et al., 2017). Exercise can also help reduce stress responsivity (cf. Zschucke et al., 2015). The efficacy of these BED treatment options should be determined in future clinical studies.

## Ultimate-Level Prevention of Eating Disorders

The above treatments focus on the proximate mechanisms underlying eating disorders; yet, for any treatments to have substantial long-term efficacy, the ultimate causation behind eating disorders should also be addressed. Media literacy programmes could constitute an effective preventative measure for eating disorders (Li et al., 2014) insofar as idealized images of slim women and muscular men in the media over-activate intrasexual competitive motives (Boothroyd et al., 2016; Borau and Bonnefon, 2017; Saunders and Eaton, 2018), and insofar as it is difficult for young people to reduce their overall exposure to media. Media literacy programmes aim to make participants informed consumers of media. They include psychoeducational components and show how photo editing software is used to make images look more “perfect” (Li et al., 2014). Media literacy programs are based on the assumption that by providing young people with facts about advertising and media images, they would be less susceptible to internalizing thin body ideals and less concerned with their weight: as a consequence, behaviors associated with eating disorders would be reduced (Li et al., 2014).

Media literacy programs have been effective in reducing cognitive states associated with eating disorders. A study on undergraduate students reported that a 4-week media literacy intervention decreased body dissatisfaction and internalization of sociocultural ideals of thinness (Watson and Vaugn, 2006). Another study found that participating in an eight-session media literacy program reduced shape, weight and dieting concerns and body dissatisfaction in adolescent girls – the effect persisted at a 30-month follow-up, suggesting a long-term improvement (Wilksch and Wade, 2009). In addition to media literacy, dissonance-based approaches, in which participants are trained to take a counter-attitudinal stance against thin beauty ideals, have been effective in reducing behaviors and thoughts associated with eating disorders (Stice et al., 2001, 2006; Becker et al., 2006; Yager and O’Dea, 2008). Li et al. (2014) suggested that a combination of media literacy programs and the dissonance approach might be an effective way to prevent eating disorders in adolescents.

## Conclusion

Converging evidence indicates that eating disorders are often maladaptive responses to intrasexual competition for thinness. The mismatch hypothesis outlined in section “The Mismatch Hypothesis of Eating Disorders” further recognizes that the evolutionarily novel conditions of food abundance and sedentary lifestyle give rise to an *adaptive metaproblem* in which psychological mechanisms of food intake clash with mating-related psychological mechanisms. The large-scale prevalence of eating disorders in contemporary humans is thus an evolutionary novelty: members of no other species are known to *starve themselves to death* because of food abundance. This is a striking realization for evolutionarily informed psychiatry.

The evidence reviewed in this article has led us to suggest that rather than conceptualizing eating disorders as discrete conditions, they should be viewed on a continuum. According to the psychoneuroimmunological model that we presented in this article, variation in eating disorders may arise from individual differences in gut microbiota and stress responsivity (Figure 2), which influence neuroinflammation and the serotonergic system (Figure 1). Our synthetic model provides answers to four persistent questions: (1) why diagnostic symptoms and associated behaviors substantially overlap across the range of eating disorders, (2) why diagnosing eating disorders is challenging, (3) why patient diagnoses may shift between eating disorders over time and (4) why does AN exist in two forms: fat-phobic AN and non-fat-phobic AN. Future empirical work led by this model is expected to further develop the prevailing biopsychosocial understanding of eating disorders.

This review article suggests that differences between eating disorders might be mediated by variation and covariation in stress responsivity and neuroinflammation caused by chronic stress. When the degree of stress and (subsequently) stress responsivity and neuroinflammation change, the model predicts that a patient’s symptoms and eating disorder diagnosis change accordingly (Figure 2). The evidence that we have reviewed suggests that the difference between BN and AN phenotypes arises from the degree of neuroinflammation caused by chronic stress, with AN patients having stronger neuroinflammation than BN patients. Thus, a patient’s position in the eating disorder continuum (Figure 2) is determined by their stress responsivity and neuroinflammation, both of which are influenced by the chronicity of their stress.

In light of the evidence reviewed in this article, it is plausible that neuroinflammation maintains the obsession to lose weight in patients with eating disorders, being highest in AN and lowest in BED patients. BED patients do not purge, suggesting weaker obsession to lose weight than in BN patients. This obsession is mediated by pervasive mental intrusions about food, body weight, diet, physical exercise and appearance, as well as OCD-like behaviors targeted to address these issues. We hypothesize that the stronger the neuroinflammation is in AN patients, the stronger is their obsession to lose weight and fear of weight gain, and the more persistent and extreme are their OCD-like behaviors (Figure 1). Further evidence for our model is given by findings on the efficacy of olanzapine (Dold et al., 2015; Himmerich et al., 2017) and zinc as treatments for AN (Safai-Kutti and Kutti, 1986; Safai-Kutti, 1990; Birmingham et al., 1994; Birmingham and Gritzner, 2006). After all, both are known to have anti-inflammatory properties. Future research could be conducted on the effectiveness of another anti-inflammatory agent, minocycline, as a treatment option for AN and BN (cf. Esalatmanesh et al., 2016).

Since converging (though indirect) evidence indicates that patients with anorexia nervosa have neuroinflammation, positron emission tomography scanning (PET) studies are needed to provide additional support for the hypothesis that neuroinflammation is a biological mechanism that underlies the spectrum of eating disorders. In addition, follow-up studies in which stress hormone levels, stress responsivity, serotonin levels, neuroinflammation and the composition of gut microbiota are measured from patients *in the course* of eating disorder(s) would reveal whether symptoms change according to predictions that arise from the model. Ultimately, we hope that the evolutionary psychoneuroimmunological model presented here will promote further empirical work, provide substantial improvements in therapeutic treatments and drugs for eating disorders and eventually prove its practical utility for the millions of people who lead lives severely debilitated by eating disorders.

## Author Contributions

MR drafted the manuscript. MR conceptualized the psychoneuroimmunological model. SL conceptualized the mismatch hypothesis. SL, TK, and IK critically reviewed the manuscript for intellectual content. MR and SL prepared the figures. All authors contributed to and approved the final version of the manuscript.

## Conflict of Interest

The authors declare that the research was conducted in the absence of any commercial or financial relationships that could be construed as a potential conflict of interest.

## References

[B1] Abed R. Mehta S. Figueredo A. J. Aldridge S. Balson H. Meyer C. (2012). Eating disorders and intrasexual competition: testing an evolutionary hypothesis among young women. *Sci. World J.* 2012:290813. 10.1100/2012/290813 22566764 PMC3330742

[B2] Abed R. T. (1998). The sexual competition hypothesis for eating disorders. *Br. J. Med. Psychol.* 71 525–547. 10.1111/j.2044-8341.1998.tb01007.x 9875960

[B3] Adams T. G. Kelmendi B. Brake C. Gruner P. Badour C. (2018). The role of stress in the pathogenesis and maintenance of obsessive-compulsive disorder. *Chronic Stress* 2:2470547018758043. 10.1177/2470547018758043 29527593 PMC5841259

[B4] Adell A. Garciamarquez C. Armario A. Gelpi E. (1988). Chronic stress increases serotonin and noradrenaline in rat brain and sensitizes their responses to a further acute stress. *J. Neurochem.* 50 1678–1681. 10.1111/j.1471-4159.1988.tb02462.x 2453609

[B5] Al-Shawaf L. (2016). The evolutionary psychology of hunger. *Appetite* 105 591–595. 10.1016/j.appet.2016.06.021 27328100

[B6] Alboni S. Di Bonaventura M. V. M. Benatti C. Giusepponi M. E. Brunello N. Cifani C. (2017). Hypothalamic expression of inflammatory mediators in an animal model of binge eating. *Behav. Brain Res.* 320 420–430. 10.1016/j.bbr.2016.10.044 27984048

[B7] Alonso-Pedrero L. Bes-Rastrollo M. Marti A. (2019). Effects of antidepressant and antipsychotic use on weight gain: a systematic review. *Obes. Rev.* 10.1111/obr.12934 [Epub ahead of print]. 31524318

[B8] Altman S. E. Shankman S. A. (2009). What is the association between obsessive-compulsive disorder and eating disorders? *Clin. Psychol. Rev.* 29 638–646. 10.1016/j.cpr.2009.08.001 19744759

[B9] American Psychiatric Association, (2013). *Diagnostic and Statistical Manual of Mental Disorders : DSM-5*, 5th Edn Washington, D.C: American Psychiatric Publishing.

[B10] Anderluh M. B. Tchanturia K. Rabe-Hesketh S. Treasure J. (2003). Childhood obsessive-compulsive personality traits’ in adult women with eating disorders: defining a broader eating disorder phenotype. *Am. Psychiatry* 160 242–247. 10.1176/appi.ajp.160.2.242 12562569

[B11] Andrews T. M. Lukaszewski A. W. Simmons Z. L. Bleske-Rechek A. (2017). Cue-based estimates of reproductive value explain women’s body attractiveness. *Evol. Hum. Behav.* 38 461–467. 10.1016/j.evolhumbehav.2017.04.002

[B12] Anttila V. Bulik-Sullivan B. Finucane H. K. Walters R. K. Bras J. Duncan L. (2018). Analysis of shared heritability in common disorders of the brain. *Science* 360 eaa8757. 10.1126/science.aap8757 29930110 PMC6097237

[B13] Arcelus J. Mitchell A. J. Wales J. Nielsen S. (2011). Mortality rates in patients with anorexia nervosa and other eating disorders a meta-analysis of 36 studies. *Arch. Gen. Psychiatry* 68 724–731. 10.1001/archgenpsychiatry.2011.74 21727255

[B14] Arnold C. (2013). *Decoding Anorexia: How Breakthroughs in Science Offer Hope for Eating Disorders.* New York, NY: Routledge/Taylor & Francis Group.

[B15] Arthur-Cameselle J. Sossin K. Quatromoni P. (2017). A qualitative analysis of factors related to eating disorder onset in female collegiate athletes and non-athletes. *Eat. Disord.* 25 199–215. 10.1080/10640266.2016.1258940 27897463

[B16] Attwells S. Setiawan E. Wilson A. A. Rusjan P. M. Mizrahi R. Miler L. (2017). Inflammation in the neurocircuitry of obsessive compulsive disorder. *Biol. Psychiatry* 81 S97–S97. 10.1001/jamapsychiatry.2017.1567 28636705 PMC5710556

[B17] Bacaltchuk J. Hay P. (2003). Antidepressants versus placebo for people with bulimia nervosa. *Cochrane Database Syst. Rev.* 4:CD003391. 10.1002/14651858.CD003391 14583971

[B18] Bailer U. F. Kaye W. H. (2011). “Serotonin: Imaging Findings in Eating Disorders,” in *Behavioral Neurobiology of Eating Disorders*, Vol. 6 eds Adan R. A. H. Kaye W. H. (Berlin: Springer-Verlag Berlin), 59–79. 10.1007/7854_2010_78 PMC5957507 21243470

[B19] Bártová K. Štěrbová Z. Varella M. A. C. Valentova J. V. (2020). Femininity in men and masculinity in women is positively related to sociosexuality. *Pers. Individ. Differ.* 152:109575 10.1016/j.paid.2019.109575

[B20] Bastiani A. M. Altemus M. Pigott T. A. Rubenstein C. Weltzin T. E. Kaye W. H. (1996). Comparison of obsessions and compulsions in patients with anorexia nervosa and obsessive compulsive disorder. *Biol. Psychiatry* 39 966–969. 10.1016/0006-3223(95)00306-1 9162209

[B21] Bateson P. Laland K. N. (2013). Tinbergen’s four questions: an appreciation and an update. *Trends Ecol. Evol.* 28 712–718. 10.1016/j.tree.2013.09.013 24144467

[B22] Baumeister R. F. Reynolds T. Winegard B. Vohs K. D. (2017). Competing for love: applying sexual economics theory to mating contests. *J. Econ. Psychol.* 63 230–241. 10.1016/j.joep.2017.07.009

[B23] Becker C. B. Smith L. M. Ciao A. C. (2006). Peer-facilitated eating disorder prevention: a randomized effectiveness trial of cognitive dissonance and media advocacy. *J. Couns. Psychol.* 53 550–555. 10.1037/0022-0167.53.4.550

[B24] Behl A. Swami G. Sircar S. S. Bhatia M. S. Banerjee B. D. (2010). Relationship of possible stress-related biochemical markers to oxidative/antioxidative status in obsessive-compulsive disorder. *Neuropsychobiology* 61 210–214. 10.1159/000306591 20389131

[B25] Bekhbat M. Neigh G. N. (2018). Sex differences in the neuro-immune consequences of stress: focus on depression and anxiety. *Brain Behav. Immun.* 67 1–12. 10.1016/j.bbi.2017.02.006 28216088 PMC5559342

[B26] Berge J. M. Loth K. Hanson C. Croll-Lampert J. Neumark-Sztainer D. (2012). Family life cycle transitions and the onset of eating disorders: a retrospective grounded theory approach. *J. Clin. Nursing* 21 1355–1363. 10.1111/j.1365-2702.2011.03762.x 21749510 PMC3207010

[B27] Berk M. Williams L. J. Jacka F. N. O’Neil A. Pasco J. A. Moylan S. (2013). So depression is an inflammatory disease, but where does the inflammation come from? *BMC Med.* 11:200. 10.1186/1741-7015-11-200 24228900 PMC3846682

[B28] Berlin H. A. Koran L. M. Jenike M. A. Shapira N. A. Chaplin W. Pallanti S. (2011). Double-blind, placebo-controlled trial of topiramate augmentation in treatment-resistant obsessive-compulsive disorder. *J. Clin. Psychiatry* 72 716–721. 10.4088/JCP.09m05266gre 20816027

[B29] Birmingham C. Gritzner S. (2006). How does zinc supplementation benefit anorexia nervosa? *Eat. Weight Disord.* 11 e109–e111. 10.1007/bf03327573 17272939

[B30] Birmingham C. L. Goldner E. M. Bakan R. (1994). Controlled trial of zinc supplementation in anorexia nervosa. *Int. J. Eat. Disord.* 15 251–255. 8199605

[B31] Birmingham C. L. Touyz S. Harbottle J. (2009). Are anorexia nervosa and bulimia nervosa separate disorders? Challenging the ‘transdiagnostic’. *Theor. Eat. Disord. Eur. Eat. Disord. Rev.* 17 2–13. 10.1002/erv.896 18781580

[B32] Blinder B. J. Cumella E. J. Sanathara V. A. (2006). Psychiatric comorbidities of female inpatients with eating disorders. *Psychosom. Med.* 68 454–462. 10.1097/01.psy.0000221524.77675.f5 16738079

[B33] Bodell L. P. Keel P. K. (2010). Current treatment for anorexia nervosa: efficacy, safety, and adherence. *Psychol. Res. Behav. Manag.* 3 91–108. 10.2147/PRBM.S13814 22110333 PMC3218763

[B34] Boggiano M. A. Chandler P. C. Viana J. B. Oswald K. D. Maldonado C. R. Wauford P. K. (2005). Combined dieting and stress evoke exaggerated responses to opioids in binge-eating rats. *Behav. Neurosci.* 119 1207–1214. 10.1037/0735-7044.119.5.1207 16300427

[B35] Boothroyd L. G. Jucker J. L. Thornborrow T. Jamieson M. A. Burt D. M. Barton R. A. (2016). Television exposure predicts body size ideals in rural Nicaragua. *Br. J. Psychol.* 107 752–767. 10.1111/bjop.12184 26910312

[B36] Borau S. Bonnefon J. (2017). The imaginary intrasexual competition: advertisements featuring provocative female models trigger women to engage in indirect aggression. *J. Bus. Ethics* 157:45 10.1007/s10551-017-3643-y

[B37] Bould H. De Stavola B. Magnusson C. Micali N. Dal H. Evans J. (2016). The influence of school on whether girls develop eating disorders. *Int. J. Epidemiol.* 45 480–488. 10.1093/ije/dyw037 27097749 PMC4864880

[B38] Bovet J. (2019). Evolutionary theories and men’s preferences for women’s waist-to-hip ratio: which hypotheses remain? *Front. Psychol.* 10:1221. 10.3389/fpsyg.2019.01221 31244708 PMC6563790

[B39] Bravo J. A. Forsythe P. Chew M. V. Escaravage E. Savignac H. M. Dinan T. G. (2011). Ingestion of Lactobacillus strain regulates emotional behavior and central GABA receptor expression in a mouse via the vagus nerve. *Proc. Natl. Acad. Sci. U.S.A.* 108 16050–16055. 10.1073/pnas.1102999108 21876150 PMC3179073

[B40] Brewerton T. D. George M. S. (1993). Is migraine related to the eating disorders? *Int. J. Eat. Disord.* 14 75–79. 10.1002/1098-108x(199307)14:1<75::aid-eat2260140110>3.0.co;2-d 8339102

[B41] Brewerton T. D. George M. S. Harden R. N. (1993). Migraine and the eating disorders. *Psychiatry Res.* 46 201–202. 10.1016/0165-1781(93)90020-h 8483977

[B42] Brockmeyer T. Friederich H. Schmidt U. (2017). Advances in the treatment of anorexia nervosa: a review of established and emerging interventions. *Psychol. Med.* 11 1–37. 10.1017/S0033291717002604 28889819

[B43] Bulik C. M. Sullivan P. F. Fear J. Pickering A. (1997). Predictors of the development of bulimia nervosa in women with anorexia nervosa. *J. Nerv. Ment. Dis.* 185 704–707. 10.1097/00005053-199711000-00009 9368548

[B44] Burton A. Abbott M. (2019). Processes and pathways to binge eating: development of an integrated cognitive and behavioural model of binge eating. *J. Eat. Disord.* 7:18. 10.1186/s40337-019-0248-0 31183111 PMC6554957

[B45] Bustamante A. C. Aiello A. E. Galea S. Ratanatharathorn A. Noronha C. Wildman D. E. (2016). Glucocorticoid receptor DNA methylation, childhood maltreatment and major depression. *J. Affect. Disord.* 206 181–188. 10.1016/j.jad.2016.07.038 27475889 PMC5077661

[B46] Butovskaya M. Sorokowska A. Karwowski M. Sabiniewicz A. Fedenok J. Dronova D. (2017). Waist-to-hip ratio, body-mass index, age and number of children in seven traditional societies. *Sci. Rep.* 7:1622. 10.1038/s41598-017-01916-9 28487573 PMC5431669

[B47] Calcia M. A. Bonsall D. R. Bloomfield P. S. Selvaraj S. Barichello T. Howes O. D. (2016). Stress and neuroinflammation: a systematic review of the effects of stress on microglia and the implications for mental illness. *Psychopharmacology* 233 1637–1650. 10.1007/s00213-016-4218-9 26847047 PMC4828495

[B48] Calzo J. Austin S. Micali N. (2018). Sexual orientation disparities in eating disorder symptoms among adolescent boys and girls in the UK. *Eur. Child Adolesc. Psyhiatry* 27 1483–1490. 10.1007/s00787-018-1145-9 29550905 PMC6141356

[B49] Carlat D. J. Camargo C. A. Herzog D. B. (1997). Eating disorders in males: a report on 135 patients. *Am. J. Psychiatry* 154 1127–1132. 10.1176/ajp.154.8.1127 9247400

[B50] Caslini M. Bartoli F. Crocamo C. Dakanalis A. Clerici M. Carra G. (2016). Disentangling the association between child abuse and eating disorders: a systematic review and meta-analysis. *Psychosom. Med.* 78 79–90. 10.1097/psy.0000000000000233 26461853

[B51] Cassidy E. Allsopp M. Williams T. (1999). Obsessive compulsive symptoms at initial presentation of adolescent eating disorders. *Eur. Child Adolesc. Psychiatry* 8 193–199. 10.1007/s007870050129 10550701

[B52] Castellini G. Lo Sauro C. Ricca V. Rellini A. H. (2017). Body esteem as a common factor of a tendency toward binge eating and sexual dissatisfaction among women: the role of dissociation and stress response during sex. *J. Sex. Med.* 14 1036–1045. 10.1016/j.jsxm.2017.06.001 28666657

[B53] Caudle H. Pang C. Mancuso S. Castle D. Newton R. (2015). A retrospective study of the impact of DSM-5 on the diagnosis of eating disorders in Victoria, Australia. *J. Eat. Disord.* 3:35. 10.1186/s40337-015-0072-0 26543558 PMC4634739

[B54] Ceccarini J. Weltens N. Ly H. G. Tack J. Van Oudenhove L. Van Laere K. (2016). Association between cerebral cannabinoid 1 receptor availability and body mass index in patients with food intake disorders and healthy subjects: a F-18 MK-9470 PET study. *Trans. Psychiatry* 6:8. 10.1038/tp.2016.118 27404285 PMC5545708

[B55] Cederlof M. Thornton L. M. Baker J. Lichtenstein P. Larsson H. Ruck C. (2015). Etiological overlap between obsessive-compulsive disorder and anorexia nervosa: a longitudinal cohort, multigenerational family and twin study. *World Psychiatry* 14 333–338. 10.1002/wps.20251 26407789 PMC4592656

[B56] Chakravarthy M. V. Booth F. W. (2004). Eating, exercise, and “thrifty” genotypes: connecting the dots toward an evolutionary understanding of modern chronic diseases. *J. Appl. Physiol.* 96 3–10. 10.1152/japplphysiol.00757.2003 14660491

[B57] Clarke G. Grenham S. Scully P. Fitzgerald P. Moloney R. D. Shanahan F. (2013). The microbiome-gut-brain axis during early life regulates the hippocampal serotonergic system in a sex-dependent manner. *Mol. Psychiatry* 18 666–673. 10.1038/mp.2012.77 22688187

[B58] Corbett S. Courtiol A. Lummaa V. Moorad J. Stearns S. (2018). The transition to modernity and chronic disease: mismatch and natural selection. *Nat. Rev. Genet.* 19 419–430. 10.1038/s41576-018-0012-3 29743650

[B59] Crisp A. (1983). “Some aspects of the psychopathology of anorexia nervosa,” in *Anorexia Nervosa: Recent Developments in Research*, eds Darby P. Garfinkel P. Garner D. Coscina D. (New York, NY: Guildford Press.), 15–28.

[B60] Cuesto G. Everaerts C. Leon L. G. Acebes A. (2017). Molecular bases of anorexia nervosa, bulimia nervosa and binge eating disorder: shedding light on the darkness. *J. Neurogenet.* 31 266–287. 10.1080/01677063.2017.1353092 28762842

[B61] D’Andrea G. Ostuzzi R. Francesconi F. Musco F. Bolner A. d’Onofrio F. (2009). Migraine prevalence in eating disorders and pathophysiological correlations. *Neurol. Sci.* 30 S55–S59. 10.1007/s10072-009-0070-6 19415427

[B62] Dalley J. W. Roiser J. P. (2012). Dopamine, serotonin and impulsivity. *Neuroscience* 215 42–58. 10.1016/j.neuroscience.2012.03.065 22542672

[B63] Dalton B. Bartholdy S. Robinson L. Solmi M. Ibrahim M. A. A. Breen G. (2018). A meta-analysis of cytokine concentrations in eating disorders. *J. Psychiatric Res.* 103 252–264. 10.1016/j.jpsychires.2018.06.002 29906710

[B64] Dantzer R. (2009). Cytokine, sickness behavior, and depression. *Immunol. Allergy Clin. North Am.* 29 247–264. 10.1016/j.iac.2009.02.002 19389580 PMC2740752

[B65] Davis H. Attia E. (2017). Pharmacotherapy of eating disorders. *Curr. Opin. Psychiatry* 30 452–457. 10.1097/yco.0000000000000358 28806268 PMC8221404

[B66] de Clercq N. C. Frissen M. N. Davids M. Groen A. K. Nieuwdorp M. (2019). Weight gain after fecal microbiota transplantation in a patient with recurrent underweight following clinical recovery from anorexia nervosa. *Psychother. Psychosom.* 88 58–60. 10.1159/000495044 30625497

[B67] Del Giudice M. Ellis B. J. Shirtcliff E. A. (2011). The adaptive calibration model of stress responsivity. *Neurosci. Biobehavi. Rev.* 35 1562–1592. 10.1016/j.neubiorev.2010.11.007 21145350 PMC3068241

[B68] Del Zotto M. Pegna A. J. (2017). Electrophysiological evidence of perceived sexual attractiveness for human female bodies varying in waist-to-hip ratio. *Cogn. Affect. Behav. Neurosci.* 17 577–591. 10.3758/s13415-017-0498-8 28315140

[B69] Dignon A. Beardsmore A. Spain S. Kuan A. (2006). ‘Why i won’t eat’ - Patient testimony from 15 anorexics concerning the cause of their disorder. *J. Health Psychol.* 11 942–956. 10.1177/1359105306069097 17035265

[B70] Dold M. Aigner M. Klabunde M. Treasure J. Kasper S. (2015). Second-Generation antipsychotic drugs in anorexia nervosa: a meta-analysis of randomized controlled trials. *Psychother. Psychosom.* 84 110–116. 10.1159/000369978 25722106

[B71] Esalatmanesh S. Abrishami Z. Zeinoddini A. Rahiminejad F. Sadeghi M. Najarzadegan M. R. (2016). Minocycline combination therapy with fluvoxamine in moderate-to-severe obsessive-compulsive disorder: a placebo-controlled, double-blind, randomized trial. *Psychiatry Clin. Neurosci.* 70 517–526. 10.1111/pcn.12430 27488081

[B72] Evans G. W. Fuller-Rowell T. E. (2013). Childhood poverty, chronic stress, and young adult working memory: the protective role of self-regulatory capacity. *Devel. Sci.* 16 688–696. 10.1111/desc.12082 24033574

[B73] Faer L. M. Hendriks A. Abed R. T. Figueredo A. J. (2005). The evolutionary psychology of eating disorders: female competition for mates or for status? *Psychol. Psychother. Theory Res. Pract.* 78 397–417. 10.1348/147608305x42929 16259854

[B74] Fairburn C. G. (2008). *Cognitive Behavior Therapy and Eating Disorders.* New York, NY: Guilford Press.

[B75] Farook J. M. Lewis B. Littleton J. M. Barron S. (2009). Topiramate attenuates the stress-induced increase in alcohol consumption and preference in male C57BL/6J mice. *Physiol. Behav.* 96 189–193. 10.1016/j.physbeh.2008.08.011 18786555

[B76] Findley D. B. Leckman J. F. Katsovich L. Lin H. Zhang H. Grantz H. (2003). Development of the yale children’s global stress index (YCGSI) and its application in children and adolescents with Tourette’s syndrome and obsessive-compulsive disorder. *J. Am. Acad. Child Adolesc. Psychiatry* 42 450–457. 10.1097/01.chi.0000046816.95464.ef 12649632

[B77] Fletcher P. J. Sinyard J. Higgins G. A. (2010). Genetic and pharmacological evidence that 5-HT2C receptor activation, but not inhibition, affects motivation to feed under a progressive ratio schedule of reinforcement. *Pharmacol. Biochem. Behav.* 97 170–178. 10.1016/j.pbb.2010.07.002 20624416

[B78] Foster J. A. Rinaman L. Cryan J. F. (2017). Stress & the gut-brain axis: regulation by the microbiome. *Neurobiol. Stress* 7 124–136. 10.1016/j.ynstr.2017.03.001 29276734 PMC5736941

[B79] Frank G. K. W. DeGuzman M. C. Shott M. E. (2019). Motivation to eat and not to eat - The psycho-biological conflict in anorexia nervosa. *Physiol. Behav.* 206 185–190. 10.1016/j.physbeh.2019.04.007 30980856 PMC6520121

[B80] Frederick D. A. Haselton M. G. (2007). Why is muscularity sexy? Tests of the fitness indicator hypothesis. *Personal. Soc. Psychol. Bull.* 33 1167–1183. 10.1177/0146167207303022 17578932

[B81] Furnham A. Baguma P. (1994). Cross-cultural differences in the evaluation of male and female body shapes. *Int,. J. Eat. Disord.* 15 81–89. 10.1002/1098-108x(199401)15:1<81::aid-eat2260150110>3.0.co;2-d 8124330

[B82] Galusca B. Sigaud T. Costes N. Redoute J. Massoubre C. Estour B. (2014). Wide impairment of cerebral serotoninergic activity but inter-individual heterogeneity in bulimia nervosa patients: a pilot F-18 MPPF/PET study. *World J. Biol. Psychiatry* 15 599–608. 10.3109/15622975.2014.942358 25054914

[B83] Gammoh N. Z. Rink L. (2017). Zinc in Infection and Inflammation. *Nutrients* 9:25. 10.3390/nu9060624 28629136 PMC5490603

[B84] Gao X. H. Cao Q. H. Cheng Y. Zhao D. D. Wang Z. Yang H. B. (2018). Chronic stress promotes colitis by disturbing the gut microbiota and triggering immune system response (vol 115, pg E2960, 2018). *Proc. Natl. Acad. Sci. U.S.A.* 115 E4542–E4542. 10.1073/pnas.1806622115 29531080 PMC5879702

[B85] Garcia-Garcia A. L. Meng Q. Y. Canetta S. Gardier A. M. Guiard B. P. Kellendonk C. (2017). Serotonin Signaling through prefrontal cortex 5-HT1A receptors during adolescence can determine baseline mood-related behaviors. *Cell Rep.* 18 1144–1156. 10.1016/j.celrep.2017.01.021 28147271 PMC5325088

[B86] Garcia-Soriano G. Roncero M. Perpina C. Belloch A. (2014). Intrusive thoughts in obsessive-compulsive disorder and eating disorder patients: a differential analysis. *Eur. Eat. Disord. Rev.* 22 191–199. 10.1002/erv.2285 24596069

[B87] Goldschmidt A. B. Le Grange D. Powers P. Crow S. J. Hill L. L. Peterson C. B. (2011). Eating disorder symptomatology in normal-weight vs. obese individuals with binge eating disorder. *Obesity* 19 1515–1518. 10.1038/oby.2011.24 21331066 PMC3818144

[B88] Grossmann R. E. Zughaier S. M. Liu S. Lyles R. H. Tangpricha V. (2012). Impact of vitamin D supplementation on markers of inflammation in adults with cystic fibrosis hospitalized for a pulmonary exacerbation. *Eur. J. Clin. Nutrit.* 66 1072–1074. 10.1038/ejcn.2012.82 22805498 PMC3638806

[B89] Guisinger S. (2003). Adapted to flee famine: adding an evolutionary perspective on anorexia nervosa. *Psychol. Rev.* 110 745–761. 10.1037/0033-295x.110.4.745 14599241

[B90] Hafstrom I. Ringertz B. Gyllenhammar H. Palmblad J. Harmsringdahl M. (1988). Effects of fasting on disease activity, neutrophil function, fatty acid composition, and leukotriene biosynthesis in patients with rheumatoid arthritis. *Arthritis Rheum.* 31 585–592. 10.1002/art.1780310502 2837251

[B91] Hagan M. M. Chandler P. C. Wauford P. K. Rybak R. J. Oswald K. D. (2003). The role of palatable food and hunger as trigger factors in an animal model of stress induced binge eating. *Int. J. Eat. Disord.* 34 183–197. 10.1002/eat.10168 12898554

[B92] Hagan M. M. Wauford P. K. Chandler P. C. Jarrett L. A. Rybak R. J. Blackburn K. (2002). A new animal model of binge eating: key synergistic role of past caloric restriction and stress. *Physiol. Behav.* 77 45–54. 10.1016/s0031-9384(02)00809-0 12213501

[B93] Hale M. W. Shekhar A. Lowry C. A. (2012). Stress-related serotonergic systems: implications for symptomatology of anxiety and affective disorders. *Cell. Mol. Neurobiol.* 32 695–708. 10.1007/s10571-012-9827-1 22484834 PMC3378822

[B94] Haleem D. J. (2012). Serotonin neurotransmission in anorexia nervosa. *Behav. Pharmacol.* 23 478–495. 10.1097/FBP.0b013e328357440d 22854305

[B95] Haleem D. J. Haider S. (1996). Food restriction decreases serotonin and its synthesis rate in the hypothalamus. *Neuroreport* 7 1153–1156. 10.1097/00001756-199604260-00011 8817522

[B96] Halmi K. A. Tozzi F. Thornton L. M. Crow S. Fichter M. M. Kaplan A. S. (2005). The relation among perfectionism, obsessive-compulsive personality disorder and obsessive-compulsive disorder in individuals with eating disorders. *Int. J. Eat. Disord.* 38 371–374. 10.1002/eat.20190 16231356

[B97] Harrison A. Stavri P. Ormond L. McEnemy F. Akyol D. Al-Khairulla H. (2018). Cognitive remediation therapy for adolescent inpatients with severe and complex anorexia nervosa: a treatment trial. *Eur. Eat. Disord. Rev.* 26 230–240. 10.1002/erv.2584 29542258

[B98] Hay P. P. J. Bacaltchuk J. Stefano S. Kashyap P. (2009). Psychological treatments for bulimia nervosa and binging. *Cochrane Database Syst. Rev.* 7:CD000562. 10.1002/14651858.CD000562.pub3 19821271 PMC7034415

[B99] Hedman A. Breithaupt L. Hübel C. Thornton L. M. Tillander A. Norring C. (2019). Bidirectional relationship between eating disorders and autoimmune diseases. *J. Child Psychol. Psychiatry* 60 803–812. 10.1111/jcpp.12958 30178543

[B100] Heo Y. A. Duggan S. T. (2017). Lisdexamfetamine: a Review in binge eating disorder. *CNS Drugs* 31 1015–1022. 10.1007/s40263-017-0477-1 29134566

[B101] Higgins G. A. Silenieks L. B. Lau W. de Lannoy I. A. M. Lee D. K. H. Izhakova J. (2013). Evaluation of chemically diverse 5-HT2C receptor agonists on behaviours motivated by food and nicotine and on side effect profiles. *Psychopharmacology* 226 475–490. 10.1007/s00213-012-2919-2 23184281

[B102] Hilbert A. (2013). Cognitive-behavioral therapy for binge eating disorder in adolescents: study protocol for a randomized controlled trial. *Trials* 14:312. 10.1186/1745-6215-14-312 24066704 PMC3850645

[B103] Himmerich H. Au K. Dornik J. Bentley J. Schmidt U. Treasure J. (2017). Olanzapine treatment for patients with anorexia nervosa. *Can. J. Psychiatry, Revue Can. Psychiatrie* 62 506–507. 10.1177/0706743717709967 28683226 PMC5528990

[B104] Hofmeijer-Sevink M. K. van Oppen P. van Megen H. J. Batelaan N. M. Cath D. C. van der Wee N. J. A. (2013). Clinical relevance of comorbidity in obsessive compulsive disorder: the Netherlands OCD Association study. *J. Affect. Disord.* 150 847–854. 10.1016/j.jad.2013.03.014 23597943

[B105] Hoppen T. Chalder T. (2018). Childhood adversity as a transdiagnostic risk factor for affective disorders in adulthood: a systematic review focusing on biopsychosocial moderating and mediating variables. *Clin. Psychol. Rev.* 65 81–151. 10.1016/j.cpr.2018.08.002 30189342

[B106] Joy E. Kussman A. Nattiv A. (2016). 2016 update on eating disorders in athletes: a comprehensive narrative review with a focus on clinical assessment and management. *Br. J. Sports Med.* 50 154–162. 10.1136/bjsports-2015-095735 26782763

[B107] Karazsia B. T. Murnen S. K. Tylka T. L. (2017). Is body dissatisfaction changing across time? A cross-temporal meta-analysis. *Psychol. Bull.* 143 293–320. 10.1037/bul0000081 27893220

[B108] Katz R. L. Keen C. L. Litt I. F. Hurley L. S. Kellamsharrison K. M. Glader L. J. (1987). Zinc-deficiency in anorexia-nervosa. *J. Adolesc. Health* 8 400–406. 10.1016/0197-0070(87)90227-0 3312133

[B109] Kaye W. H. Barbarich N. C. Putnam K. Gendall K. A. Fernstrom J. Fernstrom M. (2003). Anxiolytic effects of acute tryptophan depletion in anorexia nervosa. *Int. J. Eat. Disord.* 33 257–267. 10.1002/eat.10135 12655621

[B110] Kaye W. H. Ebert M. H. Raleigh M. Lake C. R. (1984). Abnormalities in CNS monoamine metabolism in anorexia nervosa. *Arch. Gen. Psychiatry* 41 350–355. 6200083 10.1001/archpsyc.1984.01790150040007

[B111] Kaye W. H. Frank G. K. Meltzer C. C. Price J. C. McConaha C. W. Crossan P. J. (2001). Altered serotonin 2A receptor activity in women who have recovered from bulimia nervosa. *Am. J. Psychiatry* 158 1152–1155. 10.1176/appi.ajp.158.7.1152 11431241

[B112] Kaye W. H. Fudge J. L. Paulus M. (2009). New insights into symptoms and neurocircuit function of anorexia nervosa. *Nat. Rev. Neurosci.* 10 573–584. 10.1038/nrn2682 19603056 PMC13038070

[B113] Kaye W. H. Gwirtsman H. E. George D. T. Ebert M. H. (1991). Altered serotonin activity in anorexia nervosa after long-term weight restoration: does elevated cerebrospinal fluid 5-hydroxyindoleacetic acid level correlate with rigid and obsessive behavior? *Arch. Gen. Psychiatry* 48 556–562. 1710099 10.1001/archpsyc.1991.01810300068010

[B114] Kaye W. H. Gwirtsman H. E. George D. T. Jimerson D. C. Ebert M. H. (1988). CSF 5-HIAA concentrations in anorexia nervosa: reduced values in underweight subjects normalize after weight gain. *Biol. Psychiatry* 23 102–105. 10.1016/0006-3223(88)90113-8 2447961

[B115] Kaye W. H. Weltzin T. Hsu L. K. G. (1993). Relationship between anorexia nervosa and obsessive and compulsive behaviors. *Psychiatric Ann.* 23 365–373. 10.3928/0048-5713-19930701-07

[B116] Keel P. K. Klump K. L. Miller K. B. McGue M. Iacono W. G. (2005). Shared transmission of eating disorders and anxiety disorders. *Int. J. Eat. Disord.* 38 99–105. 10.1002/eat.20168 16134107

[B117] Keeney A. Jessop D. S. Harbuz M. S. Marsden C. A. Hogg S. Blackburn-Munro R. E. (2006). Differential effects of acute and chronic social defeat stress on hypothalamic-pituitary-adrenal axis function and hippocampal serotonin release in mice. *J. Neuroendocrinol.* 18 330–338. 10.1111/j.1365-2826.2006.01422.x 16629831

[B118] Keski-Rahkonen A. Mustelin L. (2016). Epidemiology of eating disorders in Europe: prevalence, incidence, comorbidity, course, consequences, and risk factors. *Curr. Opin. Psychiatry* 29 340–345. 10.1097/yco.0000000000000278 27662598

[B119] Keys A. (1950). *The Biology of Human Starvation.* Minneapolis: University of Minnesota Press.

[B120] Khademian M. Farhangpajouh N. Shahsanaee A. Bahreynian M. Mirshamsi M. Kelishadi R. (2014). Effects of zinc supplementation on subscales of anorexia in children: a randomized controlled trial. *Pakistan J. Med. Sci.* 30 1213–1217. 10.12669/pjms.306.6377 25674110 PMC4320702

[B121] Khani S. Tayek J. A. (2001). Cortisol increases gluconeogenesis in humans: its role in the metabolic syndrome. *Clin. Sci.* 101 739–747. 10.1042/cs20010180 11724664

[B122] King B. M. (2013). The modern obesity epidemic, ancestral hunter-gatherers, and the sensory/reward control of food intake. *Am. Psychol.* 68 88–96. 10.1037/a0030684 23244211

[B123] Klatzkin R. R. Gaffney S. Cyrus K. Bigus E. Brownley K. A. (2018). Stress-induced eating in women with binge-eating disorder and obesity. *Biol. Psychol.* 131 96–106. 10.1016/j.biopsycho.2016.11.002 27836626

[B124] Kleiman S. C. Watson H. J. Bulik-Sullivan E. C. Huh E. Y. Tarantino L. M. Bulik C. M. (2015). The intestinal microbiota in acute anorexia nervosa and during renourishment: relationship to depression. anxiety, and eating disorder psychopathology. *Psychosom. Med.* 77 969–981. 10.1097/psy.0000000000000247 26428446 PMC4643361

[B125] Klump K. L. Culbert K. M. Sisk C. L. (2017). Sex differences in binge eating: gonadal hormone effects across development. *Annu. Rev. Clin. Psychol.* 13 183–207. 10.1146/annurev-clinpsy-032816-045309 28301762

[B126] Koduah P. Paul F. Dorr J. M. (2017). Vitamin D in the prevention, prediction and treatment of neurodegenerative and neuroinflammatory diseases. *Epma J.* 8 313–325. 10.1007/s13167-017-0120-8 29209434 PMC5700019

[B127] Konuk N. Tekin I. O. Ozturk U. Atik L. Atasoy N. Bektas S. (2007). Plasma levels of tumor necrosis factor-alpha and interleukin-6 in obsessive compulsive disorder. *Med. Inflamm.* 2007:65704. 10.1155/2007/65704 PMC1847475 17497035

[B128] Krams I. Rantala M. J. Luoto S. Krama T. (2018). Fat is not just an energy store. *J. Exp. Biol.* 221 (Pt 12):jeb183756. 10.1242/jeb.183756 29934415

[B129] Krams I. A. Kecko S. Joers P. Trakimas G. Elferts D. Krams R. (2017). Microbiome symbionts and diet diversity incur costs on the immune system of insect larvae. *J. Exp. Biol.* 220 4204–4212. 10.1242/jeb.169227 28939559

[B130] Kudielka B. M. Kirschbaum C. (2005). Sex differences in HPA axis responses to stress: a review. *Biol. Psychol.* 69 113–132. 10.1016/j.biopsycho.2004.11.009 15740829

[B131] Lassek W. D. Gaulin S. (2019). Evidence supporting nubility and reproducitve value as the key to human female physical attractiveness. *Evol. Hum. Behav..* 10.1016/j.evolhumbehav.2019.05.001 [Epub ahead of print].

[B132] Lebow J. Chuy J. A. Cedermark K. Cook K. Sim L. A. (2015). The development or exacerbation of eating disorder symptoms after topiramate initiation. *Pediatrics* 135 E1312–E1316. 10.1542/peds.2014-3413 25847809

[B133] Legenbauer T. Thiemann P. Vocks S. (2014). Body image disturbance in children and adolescents with eating disorders current evidence and future directions. *Z. Kinder Jugendpsychiat. Psychother.* 42 51–59. 10.1024/1422-4917/a000269 24365963

[B134] Leone A. Martinez-Gonzalez M. A. Lahortiga-Ramos F. Santos P. M. Bertoli S. Battezzati A. (2018). Adherence to the mediterranean dietary pattern and incidence of anorexia and bulimia nervosa in women: the SUN cohort. *Nutrition* 54 19–25. 10.1016/j.nut.2018.02.008 29704863

[B135] Lewis D. M. G. Al-Shawaf L. Conroy-Beam D. Asao K. Buss D. M. (2017). Evolutionary psychology: a how-to guide. *Am. Psychol.* 72 353–373. 10.1037/a0040409 28481582

[B136] Li N. P. Smith A. R. Griskevicius V. Cason M. J. Bryan A. (2010). Intrasexual competition and eating restriction in heterosexual and homosexual individuals. *Evol. Hum. Behav.* 31 365–372. 10.1016/j.evolhumbehav.2010.05.004 20835352 PMC2935594

[B137] Li N. P. Smith A. R. Yong J. C. Brown T. A. (2014). “Intrasexual Competition and Other Theories of Eating Restriction,” in *Evolutionary Perspectives on Human Sexual Psychology and Behavior. Evolutionary Psychology*, eds Weekes-Shackelford V. Shackelford T. (New York, NY: Springer). 10.1016/j.evolhumbehav.2010.05.004

[B138] Li N. P. van Vugt M. Colarelli S. M. (2018). The evolutionary mismatch hypothesis: implications for psychological science. *Curr. Dir. Psychol. Sci.* 27 38–44. 10.1177/0963721417731378

[B139] Liang S. Wu X. L. Jin F. (2018). Gut-brain psychology: rethinking psychology from the microbiota-gut-brain axis. *Front. Integr. Neurosci.* 12:24. 10.3389/fnint.2018.00033 30271330 PMC6142822

[B140] Lindeberg S. (2010). *Food and Western Disease: Health and Nutrition From an Evolutionary Perspective*. Ames: Wiley-Blackwell.

[B141] Lissemore J. I. Sookman D. Gravel P. Berney A. Barsoum A. Diksic M. (2018). Brain serotonin synthesis capacity in obsessive-compulsive disorder: effects of cognitive behavioral therapy and sertraline. *Trans. Psychiatry* 8:82. 10.1038/s41398-018-0128-4 29666372 PMC5904107

[B142] Love H. Sulikowski D. (2018). Of meat and men: sex differences in implicit and explicit attitudes towards meat. *Front. Psychol.* 9:559. 10.3389/fpsyg.2018.00559 29731733 PMC5920154

[B143] Lukens J. R. Gurung P. Vogel P. Johnson G. R. Carter R. A. McGoldrick D. J. (2014). Dietary modulation of the microbiome affects autoinflammatory disease. *Nature* 516 246–249. 10.1038/nature13788 25274309 PMC4268032

[B144] Luoto S. Karlsson H. Krams I. Rantala M. (2018). Depression subtyping based on evolutionary psychiatry: from reactive short-term mood change to depression. *Brain Behav. Immun.* 69:630. 10.1016/j.bbi.2017.10.012 29203424

[B145] Luoto S. (2019a). An updated theoretical framework for human sexual selection: from ecology, genetics, and life history to extended phenotypes. *Adapt. Hum. Behav. Physiol.* 5 48–102. 10.1007/s40750-018-0103-6

[B146] Luoto S. (2019b). Response to commentaries: life history genetics, fluid intelligence, and extended phenotypes. *Adapt. Hum. Behav. Physiol.* 5 112–115. 10.1007/s40750-019-0109-8

[B147] Luoto S. Krams I. Rantala M. J. (2019a). A life history approach to the female sexual orientation spectrum: evolution, development, causal mechanisms, and health. *Arch. Sex. Beha.* 48 1273–1308. 10.1007/s10508-018-1261-0 30229521

[B148] Luoto S. Krams I. Rantala M. J. (2019b). Response to commentaries: life history evolution, causal mechanisms, and female sexual orientation. *Arch. Sex. Behav.* 48 1335–1347. 10.1007/s10508-019-1439-0 31119422

[B149] Machado P. P. P. Goncalves S. Hoek H. W. (2013). DSM-5 reduces the proportion of ednos cases: evidence from community samples. *Int. J. Eat. Disord.* 46 60–65. 10.1002/eat.22040 22815201

[B150] Mack I. Cuntz U. Gramer C. Niedermaier S. Pohl C. Schwiertz A. (2016). Weight gain in anorexia nervosa does not ameliorate the faecal microbiota, branched chain fatty acid profiles, and gastrointestinal complaints. *Sci. Rep.* 6 26752. 10.1038/srep26752 27229737 PMC4882621

[B151] Malhotra R. (2016). Understanding migraine: potential role of neurogenic inflammation. *Ann. Indian Acad. Neurol* 19 175–182. 10.4103/0972-2327.182302 27293326 PMC4888678

[B152] Mancuso S. G. Newton J. R. Bosanac P. Rossell S. L. Nesci J. B. Castle D. J. (2015). Classification of eating disorders: comparison of relative prevalence rates using DSM-IV and DSM-5 criteria. *Br. J. Psychiatry* 206 519–520. 10.1192/bjp.bp.113.143461 25745131

[B153] Mann T. Tomiyama A. J. Westling E. Lew A. M. Samuels B. Chatman J. (2007). Medicare’s search for effective obesity treatments - Diets are not the answer. *Am. Psychol.* 62 220–233. 10.1037/0003-066x.62.3.220 17469900

[B154] Marquez S. (2008). Eating disorders in sports: risk factors, health consequences, treatment and prevention. *Nutr. Hosp.* 23 183–190. 18560693

[B155] Martin J. R. Bos M. Jenck F. Moreau J. L. Mutel V. Sleight A. J. (1998). 5-HT2C receptor agonists: pharmacological characteristics and therapeutic potential. *J. Pharmacol. Exp. Ther.* 286 913–924. 9694950

[B156] Masheb R. M. Grilo C. M. White M. A. (2011). An examination of eating patterns in community women with bulimia nervosa and binge eating disorder. *Int. J. Eat. Disord.* 44 618–624. 10.1002/eat.20853 21997425 PMC3646558

[B157] Mathot K. J. Frankenhuis W. (2018). Models of pace-of-life syndromes (POLS): a systematic review. *Behav. Ecol. Sociobiol.* 73 41.

[B158] Mayhew A. J. Pigeyre M. Couturier J. Meyre D. (2018). An evolutionary genetic perspective of eating disorders. *Neuroendocrinology* 106 292–306. 10.1159/000484525 29065413

[B159] McElroy S. L. Guerdjikova A. I. Mori N. O’Melia A. M. (2012). Pharmacological management of binge eating disorder: current and emerging treatment options. *Ther. Clin. Risk Manag.* 8 219–241. 10.2147/tcrm.s25574 22654518 PMC3363296

[B160] Mealey L. (2000). Anorexia: a “losing” strategy? *Hum. Nat.* 11 105–116. 10.1007/s12110-000-1005-3 26193098

[B161] Milaneschi Y. Simmons W. K. van Rossum E. F. C. Penninx B. W. (2018). Depression and obesity: evidence of shared biological mechanisms. *Mol. Psychiatry* 24 18–33. 10.1038/s41380-018-0017-5 29453413

[B162] Mitchell J. E. Roerig J. Steffen K. (2013). Biological therapies for eating disorders. *Int. J. Eat. Disord.* 46 470–477. 10.1002/eat.22104 23658094 PMC4372845

[B163] Molina-Torres G. Rodriguez-Arrastia M. Roman P. Sanchez-Labraca N. Cardona D. (2019). Stress and the gut microbiota-brain axis. *Behav. Pharmacol.* 30 187–200. 10.1097/FBP.0000000000000478 30844962

[B164] Mowla A. Khajeian A. M. Sahraian A. Chohedri A. H. Kashkoli F. (2010). Topiramate augmentation in resistant OCD: a double-blind placebo-controlled clinical trial. *CNS Spectr.* 15 613–617. 10.1017/s1092852912000065 24726048

[B165] Mueller A. S. Pearson J. Muller C. Frank K. Turner A. (2010). Sizing up peers: adolescent girls’ weight control and social comparison in the school context. *J. Health Soc. Behav.* 51 64–78. 10.1177/0022146509361191 20420295 PMC4074007

[B166] Murray S. B. Quintana D. S. Loeb K. L. Griffiths S. Le Grange D. (2019). Treatment outcomes for anorexia nervosa: a systematic review and meta-analysis of randomized controlled trials. *Psychol. Med.* 49 535–544. 10.1017/S0033291718002088 30101734

[B167] Mustelin L. Bulik C. M. Kaprio J. Keski-Rahkonen A. (2017). Prevalence and correlates of binge eating disorder related features in the community. *Appetite* 109 165–171. 10.1016/j.appet.2016.11.032 27899295

[B168] Naisbitt C. Davies S. (2017). Starvation, exercise and the stress response. *Anaesth. Intensive Care Med.* 18 508–512. 10.1016/j.mpaic.2017.06.020

[B169] Najjar S. Pearlman D. M. Alper K. Najjar A. Devinsky O. (2013). Neuroinflammation and psychiatric illness. *J. Neuroinflamm.* 10:43. 10.1186/1742-2094-10-43 23547920 PMC3626880

[B170] Nave G. Nadler A. Dubois D. Zava D. Camerer C. Plassmann H. (2018). Single-dose testosterone administration increases men’s preference for status goods. *Nat. Commun.* 9:2433. 10.1038/s41467-018-04923-0 29970895 PMC6030157

[B171] Nettersheim J. Gerlach G. Herpertz S. Abed R. Figueredo A. Brüne M. (2018). Evolutionary psychology of eating disorders: an explorative study in patients with anorexia nervosa and bulimia nervosa. *Front. Psychol.* 9:2122. 10.3389/fpsyg.2018.02122 30429818 PMC6220092

[B172] Neumark-Sztainer D. (2005). *I’m, like, SO fat!.* New York, NY: Guilford Press.

[B173] Ni J. Shen T. C. D. Chen E. Z. Bittinger K. Bailey A. Roggiani M. (2017). A role for bacterial urease in gut dysbiosis and Crohn’s disease. *Sci. Trans. Med.* 9:eaah6888. 10.1126/scitranslmed.aah6888 29141885 PMC5808452

[B174] O’Mahony S. M. Neufeld K. A. M. Waworuntu R. V. Berg B. M. Dinan T. G. Cryan J. F. (2016). A combination of dietary prebiotics and the probiotic LGG modulate behavioural and cognitive reponses to early life stress. *Neurogastroenterol. Motil.* 28 13–13. 10.1111/j.2042-7166.2005.tb00466.x

[B175] Olguin P. Fuentes M. Gabler G. Guerdjikova A. I. Keck P. E. McElroy S. L. (2017). Medical comorbidity of binge eating disorder. *Eat. Weight Disord.* 22 13–26. 10.1007/s40519-016-0313-5 27553016

[B176] Oliver G. Wardle J. (1999). Perceived effects of stress on food choice. *Physiol. Behav.* 66 511–515. 10.1016/s0031-9384(98)00322-9 10357442

[B177] Orth U. Robins R. W. (2013). Understanding the link between low self-esteem and depression. *Curr. Dir. Psychol. Sci.* 22 455–460. 10.1177/0963721413492763

[B178] Pan W. H. Wu X. J. He Y. Hung H. C. Huang E. Y. K. Mishra P. K. (2013). Brain interleukin-15 in neuroinflammation and behavior. *Neurosci. Biobehav. Rev.* 37 184–192. 10.1016/j.neubiorev.2012.11.009 23201098 PMC3563733

[B179] Park C. Brietzke E. Rosenblat J. D. Musial N. Zuckerman H. Ragguett R. M. (2018). Probiotics for the treatment of depressive symptoms: an anti-inflammatory mechanism? *Brain Behav. Immun.* 73 115–124. 10.1016/j.bbi.2018.07.006 30009996

[B180] Partrick K. A. Chassaing B. Beach L. Q. McCann K. E. Gewirtz A. T. Huhman K. L. (2018). Acute and repeated exposure to social stress reduces gut microbiota diversity in Syrian hamsters (vol 345, pg 39, 2018). *Behavi. Brain Res.* 348 277–277. 10.1016/j.bbr.2018.03.044 29474810 PMC6246037

[B181] Pearl R. L. White M. A. Grilo C. M. (2014). Overvaluation of shape and weight as a mediator between self-esteem and weight bias internalization among patients with binge eating disorder. *Eat. Behav.* 15 259–261. 10.1016/j.eatbeh.2014.03.005 24854815 PMC4053161

[B182] Perkins S. J. Keville S. Schmidt U. Chalder T. (2005). Eating disorders and irritable bowel syndrome: is there a link? *J. Psychosom. Res.* 59 57–64. 10.1016/j.jpsychores.2004.04.375 16185999

[B183] Pinheiro R. M. C. de Lima M. N. M. Portal B. C. D. Busato S. B. Falavigna L. Ferreira R. D. (2015). Long-lasting recognition memory impairment and alterations in brain levels of cytokines and BDNF induced by maternal deprivation: effects of valproic acid and topiramate. *J. Neural Transm.* 122 709–719. 10.1007/s00702-014-1303-2 25182413

[B184] Pope H. G. Katz D. L. Hudson J. I. (1993). Anorexia nervosa and “reverse anorexia” among 108 male bodybuilders. *Compr. Psychiatry* 34 406–409. 10.1016/0010-440x(93)90066-d 8131385

[B185] Power M. L. (2012). The human obesity epidemic, the mismatch paradigm, and our modern “captive” environment. *Am. J. Hum. Biol.* 24 116–122. 10.1002/ajhb.22236 22287210

[B186] Price A. E. Anastasio N. C. Stutz S. J. Hommel J. D. Cunningham K. A. (2018). Serotonin 5-HT2c receptor activation suppresses binge intake and the reinforcing and motivational properties of high-fat food. *Front. Pharmacol.* 9:821. 10.3389/fphar.2018.00821 30100875 PMC6072841

[B187] Quinton S. J. Smith A. R. Joiner T. (2011). The 2nd to 4th digit ratio (2D:4D) and eating disorder diagnosis in women. *Personal. Individ. Differ.* 51 402–405. 10.1016/j.paid.2010.07.024 21765573 PMC3134962

[B188] Rantala M. Luoto S. Krams I. (2017). An evolutionary approach to clinical pharmacopsychology. *Psychother. Psychosom.* 86 370–371. 10.1159/000480709 29131107

[B189] Rantala M. Luoto S. Krams I. Karlsson H. (2018). Depression subtyping based on evolutionary psychiatry: proximate mechanisms and ultimate functions. *Brain, Behav. Immun.* 69 603–617. 10.1016/j.bbi.2017.10.012 29051086

[B190] Rohleder N. (2019). Stress and inflammation – the need to address the gap in the transition between acute and chronic stress effect. *Psychoneuroendocrinology* 105 164–171. 10.1016/j.psyneuen.2019.02.021 30826163

[B191] Ricordi C. Garcia-Contreras M. Farnetti S. (2015). Diet and inflammation: possible effects on immunity. chronic diseases, and life span. *J. Am. Coll. Nutrit.* 34 10–13. 10.1080/07315724.2015.1080101 26400428

[B192] Riva G. (2016). Neurobiology of anorexia nervosa: serotonin dysfunctions link self-starvation with body image disturbances through an impaired body memory. *Front. Hum. Neurosci.* 10:600. 10.3389/fnhum.2016.00600 27932968 PMC5121233

[B193] Rojo L. Conesa L. Bermudez O. Livianos L. (2006). Influence of stress in the onset of eating disorders: data from a two-stage epidemiologic controlled study. *Psychosom. Med.* 68 628–635. 10.1097/01.psy.0000227749.58726.41 16868274

[B194] Rolls B. J. (2017). Dietary energy density: applying behavioural science to weight management. *Nutrit. Bull.* 42 246–253. 10.1111/nbu.12280 29151813 PMC5687574

[B195] Rozin P. Todd P. (2015). “The evolutionary psychology of food intake and choice,” in *The handbook of Evolutionary Psychology*, ed. Buss D. (Hoboken, NJ: Wiley), 183–205.

[B196] Rubio G. Jimenez-Arriero M. A. Martinez-Gras I. Manzanares J. Palomo T. (2006). The effects of topiramate adjunctive treatment added to antidepressants in patients with resistant obsessive-compulsive disorder. *J. Clin. Psychopharmacol.* 26 341–344. 10.1097/01.jcp.0000220524.44905.9f 16702907

[B197] Safai-Kutti S. (1990). Oral zinc supplementation in anorexia nervosa. *Acta Psychiatr. Scand.* 82 14–17. 10.1111/j.1600-0447.1990.tb10747.x 2291418

[B198] Safai-Kutti S. Kutti J. (1986). Zinc supplementation in anorexia nervosa. *Am. J. Clin. Nutrit.* 44 581–582. 10.1093/ajcn/44.4.581 3766443

[B199] Saunders J. F. Eaton A. A. (2018). Snaps, selfies, and shares: how three popular social media platforms contribute to the sociocultural model of disordered eating among young women. *Cyberpsychol., Behav. Soc. Network.* 21 343–354. 10.1089/cyber.2017.0713 29883209

[B200] Sayyah M. Olapour A. Saeedabad Y. S. Parast R. Y. Malayeri A. (2012). Evaluation of oral zinc sulfate effect on obsessive-compulsive disorder: a randomized placebo-controlled clinical trial. *Nutrition* 28 892–895. 10.1016/j.nut.2011.11.027 22465904

[B201] Schmidt U. Adan R. Bohm I. Campbell I. C. Dingemans A. Ehrlich S. (2016). Eating disorders: the big issue. *Lancet Psychiatry* 3 313–315. 10.1016/s2215-0366(16)00081-x 27063378

[B202] Schmidt U. Oldershaw A. Jichi F. Sternheim L. Startup H. McIntosh V. (2012). Out-patient psychological therapies for adults with anorexia nervosa: randomised controlled trial. *Br. J. Psychiatry* 201 392–399. 10.1192/bjp.bp.112.112078 22995632

[B203] Schwensen H. F. Kan C. Treasure J. Hoiby N. Sjogren M. (2018). A systematic review of studies on the faecal microbiota in anorexia nervosa: future research may need to include microbiota from the small intestine. *Eat. Weight Disord.* 23 399–418. 10.1007/s40519-018-0499-9 29542066

[B204] Seitz J. Belheouane M. Schulz N. Dempfle A. Baines J. F. Herpertz-Dahlmann B. (2019). The impact of starvation on the microbiome and gut-brain interaction in anorexia nervosa. *Front. Endocrinol.* 10:41. 10.3389/fendo.2019.00041 30809191 PMC6379250

[B205] Sell A. Lukazsweski A. W. Townsley M. (2017). Cues of upper body strength account for most of the variance in men’s bodily attractiveness. *Proc. R. Soc. B Biol. Sci.* 284:20171819. 10.1098/rspb.2017.1819 29237852 PMC5745404

[B206] Sharon-Granit Y. Nassar A. Azab A. N. Kaplanski J. (2016). Effects of olanzapine and valproate on brain inflammation in lipopolysaccharide-treated rats. *Int. J. Neuropsychopharmacol.* 19 64–65.

[B207] Shields G. S. Moons W. G. Slavich G. M. (2017). Inflammation, self-regulation, and health: an immunologic model of self-regulatory failure. *Perspect. Psychol. Sci.* 12 588–612. 10.1177/1745691616689091 28679069 PMC5519413

[B208] Sjögren M. (2017). An update on genetic and serotoneric biomarker findings in Bulimia Nervosa. *EC Neurol.* 7 107–116.

[B209] Slavich G. M. Way B. M. Eisenberger N. I. Taylor S. E. (2010). Neural sensitivity to social rejection is associated with inflammatory responses to social stress. *Proc. Natl. Acad. Sci. U.S.A.* 107 14817–14822. 10.1073/pnas.1009164107 20679216 PMC2930449

[B210] Sohn K. (2016). Men’s revealed preferences regarding women’s ages: evidence from prostitution. *Evol. Hum. Behav.* 37 272–280. 10.1016/j.evolhumbehav.2016.01.002

[B211] Sokol M. S. (2000). Infection-triggered anorexia nervosa in children: clinical description of four cases. *J. Child Adolesc. Psychopharmacol.* 10 133–145. 10.1089/cap.2000.10.133 10933123

[B212] Sokol M. S. Gray N. S. (1997). Case study: an infection-triggered, autoimmune subtype of anorexia nervosa. *J. Am. Acad. Child Adolesc. Psychiatry* 36 1128–1133. 10.1097/00004583-199708000-00021 9256593

[B213] Solmi M. Santonastaso P. Caccaro R. Favaro A. (2013). A case of anorexia nervosa with comorbid Crohn’s disease: beneficial effects of anti-TNF-alpha therapy? *Int. J. Eat. Disord.* 46 639–641. 10.1002/eat.22153 23813727

[B214] Solmi M. Veronese N. Favaro A. Santonastaso P. Manzato E. Sergi G. (2015). Inflammatory cytokines and anorexia nervosa: a meta-analysis of cross-sectional and longitudinal studies. *Psychoneuroendocrinology* 51 237–252. 10.1016/j.psyneuen.2014.09.031 25462897

[B215] Song C. Merali Z. Anisman H. (1999). Variations of nucleus accumbens dopamine and serotonin following systemic interleukin-1, interleukin-2 or interleukin-6 treatment. *Neuroscience* 88 823–836. 10.1016/s0306-4522(98)00271-1 10363820

[B216] Song H. Fang F. Tomasson G. Arnberg F. K. Mataix-Cols D. Fernandez de la Cruz L. (2018). Association of stress-related disorders with subsequent autoimmune disease. *Jam. J. Am. Med. Assoc.* 319 2388–2400. 10.1001/jama.2018.7028 29922828 PMC6583688

[B217] Soukup V. M. Beiler M. E. Terrell F. (1990). Stress, coping style, and problem solving ability among eating-disordered inpatients. *J. Clin. Psychol.* 46 592–599. 2246365 10.1002/1097-4679(199009)46:5<592::aid-jclp2270460508>3.0.co;2-y

[B218] Sousa-Lima J. Moreira P. S. Raposo-Lima C. Sousa N. Morgado P. (2019). Relationship between obsessive compulsive disorder and cortisol: Systematic review and meta-analysis. *Eur. Neuropsychopharmacol.* 10.1016/j.euroneuro.2019.09.001 [Epub ahead of print]. 31540796

[B219] Speakman J. R. (2018). The evolution of body fatness: trading off disease and predation risk. *J. Exp. Biol.* 221 (Pt. Suppl. 1):jeb167254. 10.1242/jeb.167254 29514887

[B220] Speakman J. R. Levitsky D. A. Allison D. B. Bray M. S. de Castro J. M. Clegg D. J. (2011). Set points, settling points and some alternative models: theoretical options to understand how genes and environments combine to regulate body adiposity. *Dis. Models Mech.* 4 733–745. 10.1242/dmm.008698 22065844 PMC3209643

[B221] Stanton C. Holmes A. Chang S. Joormann J. (2018). From stress to anhedonia: molecular processes through functional circuits. *Trends Neurosci.* 42 23–42. 30327143 10.1016/j.tins.2018.09.008 PMC6344037

[B222] Starr T. B. Kreipe R. E. (2014). Anorexia nervosa and bulimia nervosa: brains. *Bones Breed. Curr. Psychiatry Rep.* 16:11. 10.1007/s11920-014-0441-4 24705938

[B223] Steiger H. Young S. N. Kin N. Koerner N. Israel M. Lageix P. (2001). Implications of impulsive and affective symptoms for serotonin function in bulimia nervosa. *Psychol. Med.* 31 85–95. 10.1017/s003329179900313x 11200963

[B224] Stevens A. Purcell R. Darling K. Eggleston M. Kennedy M. Rucklidge J. (2019). Human gut microbiome changes during a 10 week randomised control Trial for micronutrient supplementation in children with attention deficit hyperactivity disorder. *Sci. Rep.* 9:10128. 10.1038/s41598-019-46146-3 31300667 PMC6625977

[B225] Stice E. Chase A. Stormer S. Appel A. (2001). A randomized trial of a dissonance-based eating disorder prevention program. *Int. J. Eat. Disord.* 29 247–262. 10.1002/eat.1016 11262503

[B226] Stice E. Shaw H. Burton E. Wade E. (2006). Dissonance and healthy weight eating disorder prevention programs: a randomized efficacy trial. *J. Consult. Clin. Psychol.* 74 263–275. 10.1037/0022-006x.74.2.263 16649871 PMC1479305

[B227] Strober M. Freeman R. Lampert C. Diamond J. Kaye W. (2000). Controlled family study of anorexia nervosa and bulimia nervosa: evidence of shared liability and transmission of partial syndromes. *Am. J. Psychiatry* 157 393–401. 10.1176/appi.ajp.157.3.393 10698815

[B228] Succurro E. Segura-Garcia C. Ruffo M. Caroleo M. Rania M. Aloi M. (2015). Obese patients with a binge eating disorder have an unfavorable metabolic and inflammatory profile. *Medicine* 94:e2098. 10.1097/md.0000000000002098 26717356 PMC5291597

[B229] Sugino H. Futamura T. Mitsumoto Y. Maeda K. Marunaka Y. (2009). Atypical antipsychotics suppress production of proinflammatory cytokines and up-regulate interleukin-10 in lipopolysaccharide-treated mice. *Prog. Neuro Psychopharmacol. Biol. Psychiatry* 33 303–307. 10.1016/j.pnpbp.2008.12.006 19138716

[B230] Sugiyama L. (2015). *Physical Attractiveness: An Adaptationist Perspective. In*, 2nd Edn Hoboken,NJ: Wiley Online Library., 317–384.

[B231] Sullivan P. F. Agrawal A. Bulik C. M. Andreassen O. A. Borglum A. D. Breen G. (2018). Psychiatric genomics: an update and an agenda. *Am. J. Psychiatry* 175 15–27. 10.1176/appi.ajp.2017.17030283 28969442 PMC5756100

[B232] Surbey M. K. (1987). Anorexia nervosa, amenorrhea, and adaptation. *Ethol. Sociobiol.* 8 S47–S61.

[B233] Swami V. Frederick D. A. Aavik T. Alcalay L. Allik J. Anderson D. (2010). The attractive female body weight and female body dissatisfaction in 26 countries across 10 world regions: results of the international body project I. *Personal. Soc. Psychol. Bull.* 36 309–325. 10.1177/0146167209359702 20179313

[B234] Swanson S. A. Crow S. J. Le Grange D. Swendsen J. Merikangas K. R. (2011). Prevalence and correlates of eating disorders in adolescents results from the national comorbidity survey replication adolescent supplement. *Arch. Gen. Psychiatry* 68 714–723. 10.1001/archgenpsychiatry.2011.22 21383252 PMC5546800

[B235] Tasegian A. Curcio F. Dalla Ragione L. Rossetti F. Cataldi S. Codini M. (2016). Hypovitaminosis D3, leukopenia, and human serotonin transporter polymorphism in anorexia nervosa and bulimia nervosa. *Mediat. Inflamm.* 2016:8046479. 10.1155/2016/8046479 26903713 PMC4745338

[B236] Temko J. E. Bouhlal S. Farokhnia M. Lee M. R. Cryan J. F. Leggio L. (2017). The microbiota, the gut and the brain in eating and alcohol use disorders: a ‘M,nage A Trois’? *Alcohol. Alcohol.* 52 403–413. 10.1093/alcalc/agx024 28482009 PMC5860274

[B237] Thornton L. M. Mazzeo S. E. Bulik C. M. (2011). The heritability of eating disorders: methods and current findings. *Behav. Neurobiol. Eat. Disord.* 6 141–156. 10.1007/7854_2010_91 21243474 PMC3599773

[B238] Toro J. Cervera M. Osejo E. Salamero M. (1992). Obsessive-compulsive disorder in childhood and adolescence: a clinical study. *J. Child Psychol. Psychiatry* 33 1025–1037. 10.1111/j.1469-7610.1992.tb00923.x 1400685

[B239] Tortorella A. Fabrazzo M. Monteleone A. M. Steardo L. Monteleone P. (2014). The role of drug therapies in the treatment of anorexia and bulimia nervosa: a review of the literature. *J. Psychopathol. Giornale Psicopatol.* 20 50–65.

[B240] Tovee M. J. Swami V. Furnham A. Mangalparsad R. (2006). Changing perceptions of attractiveness as observers are exposed to a different culture. *Evol. Hum. Behav.* 27 443–456. 10.1016/j.evolhumbehav.2006.05.004

[B241] Turna J. Patterson B. Van Ameringen M. (2017). An update on the relationship between the gut microbiome and obsessive-compulsive disorder. *Psychiatric Ann.* 47 542–551. 10.3928/00485713-20171013-01

[B242] Tylee D. S. Sun J. Y. Hess J. L. Tahir M. A. Sharma E. Malik R. (2018). Genetic correlations among psychiatric and immune-related phenotypes based on genome-wide association data. *Am. J. Med. Genet. Part B Neuropsychiatric Genet.* 177 641–657. 10.1002/ajmg.b.32652 30325587 PMC6230304

[B243] Vaillancourt T. (2013). Do human females use indirect aggression as an intrasexual competition strategy? *Philos. Trans. R. Soc. B Biol. Sci.* 368:20130080. 10.1098/rstb.2013.0080 24167310 PMC3826209

[B244] Van Ameringen M. Mancini C. Patterson B. Bennett M. (2006). Topiramate augmentation in treatment-resistant obsessive-compulsive disorder: a retrospective, open-label case series. *Depress. Anxiety* 23 1–5. 10.1002/da.20118 16178009

[B245] van Furth E. F. van der Meer A. Cowan K. (2016). Top 10 research priorities for eating disorders. *Lancet Psychiatry* 3 706–707. 27475763 10.1016/S2215-0366(16)30147-X

[B246] Veronese N. Solmi M. Rizza W. Manzato E. Sergi G. Santonastaso P. (2015). Vitamin D status in anorexia nervosa: a meta-analysis. *Int. J. Eat. Disord.* 48 803–813. 10.1002/eat.22370 25445242

[B247] Vindas M. A. Johansen I. B. Folkedal O. Hoglund E. Gorissen M. Flik G. (2016). Brain serotonergic activation in growth-stunted farmed salmon: adaption versus pathology. *R. Soc. Open Sci.* 3:160030. 10.1098/rsos.160030 27293782 PMC4892444

[B248] Voland E. Voland R. (1989). Evolutionary biology and psychiatry: the case of anorexia nervosa. *Ethol. Sociobiol.* 10 223–240. 10.1016/0162-3095(89)90001-0

[B249] Wasser S. K. Barash D. P. (1983). Reproductive suppression among female mammals: implications for biomedicine and sexual selection theory. *Q. Rev. Biol.* 58 513–538. 10.1086/413545 6686686

[B250] Watson H. J. Yilmaz Z. Thornton L. M. Hübel C. Coleman J. R. Gaspar H. A. (2019). Genome-wide association study identifies eight risk loci and implicates metabo-psychiatric origins for anorexia nervosa. *Nat. Genet.* 51 1207–1214. 10.1038/s41588-019-0439-2 31308545 PMC6779477

[B251] Watson R. Vaugn L. (2006). Limiting the effects of the media on body image: does the length of the intervention make a difference? *Eat. Disord.* 14 384–400. 17062449 10.1080/10640260600952530

[B252] Wedell-Neergaard A. S. Lehrskov L. L. Christensen R. H. Legaard G. E. Dorph E. Larsen M. K. (2019). Exercise-induced changes in visceral adipose tissue mass are regulated by IL-6 signaling: a randomized controlled trial. *Cell Metab.* 29 844–855. 10.1016/j.cmet.2018.12.007 30595477

[B253] Weekes-Shackelford V. A. Shackelford T. K. (eds) (2014). *Evolutionary Perspectives on Human Sexual Psychology and Behavior.* Berlin: Springer.

[B254] Wells J. C. K. (2006). The evolution of human fatness and susceptibility to obesity: an ethological approach. *Biol. Rev.* 81 183–205. 10.1017/s1464793105006974 16677431

[B255] Wilksch S. M. Wade T. D. (2009). Reduction of shape and weight concern in young adolescents: a 30-month controlled evaluation of a media literacy Program. *J. Am. Acad. Child Adolesc. Psychiatry* 48 652–661. 10.1097/CHI.0b013e3181a1f559 19454921

[B256] Williams A. C. D. C. (2019). Persistence of pain in humans and other mammals. *Philos. Trans. R. Soc. B* 374:20190276. 10.1098/rstb.2019.0276 31544608 PMC6790389

[B257] Wotton C. J. James A. Goldacre M. J. (2016). Coexistence of eating disorders and autoimmune diseases: record linkage cohort study. *Int. J. Eat. Disord.* 49 663–672. 10.1002/eat.22544 27333941

[B258] Wu X. J. Hung H. C. Kastin A. J. He Y. Khan R. S. Stone K. P. (2011). Interleukin-15 affects serotonin system and exerts antidepressive effects through IL15R alpha receptor. *Psychoneuroendocrinology* 36 266–278. 10.1016/j.psyneuen.2010.07.017 20724079 PMC3015024

[B259] Yager Z. O’Dea J. A. (2008). Prevention programs for body image and eating disorders on University campuses: a review of large, controlled interventions. *Health Prom. Int.* 23 173–189. 10.1093/heapro/dan004 18263883

[B260] Yau Y. H. C. Potenza M. N. (2013). Stress and eating behaviors. *Minerva Endocrinol.* 38 255–267. 24126546 PMC4214609

[B261] Zerwas S. Larsen J. T. Petersen L. Thornton L. M. Quaranta M. Koch S. V. (2017). Eating disorders, autoimmune, and autoinflammatory disease. *Pediatrics* 140:e20162089. 10.1542/peds.2016-2089 29122972 PMC5703777

[B262] Zhang Y. Leung D. Y. M. Richers B. N. Liu Y. S. Remigio L. K. Riches D. W. (2012). Vitamin D inhibits monocyte/macrophage proinflammatory cytokine production by targeting MAPK phosphatase-1. *J. Immunol.* 188 2127–2135. 10.4049/jimmunol.1102412 22301548 PMC3368346

[B263] Zschucke E. Renneberg B. Dimeo F. Wüstenberg T. Ströhle A. (2015). The stress-buffering effect of acute exercise: evidence for HPA axis negative feedback. *Psychoneuroendocrinology* 51 414–425. 10.1016/j.psyneuen.2014.10.019 25462913

